# Transcriptomic data in tumor‐adjacent normal tissues harbor prognostic information on multiple cancer types

**DOI:** 10.1002/cam4.5864

**Published:** 2023-03-31

**Authors:** Euiyoung Oh, Hyunju Lee

**Affiliations:** ^1^ School of Electrical Engineering and Computer Science Gwangju Institute of Science and Technology Gwangju Republic of Korea; ^2^ Artificial Intelligence Graduate School Gwangju Institute of Science and Technology Gwangju Republic of Korea

**Keywords:** machine learning, survival prediction, tumor adjacent normal tissues

## Abstract

**Background:**

In identifying prognostic markers in cancer, the roles of tumor‐adjacent normal tissues are often confined to drawing expression differences between tumor and normal tissues rather than being treated as the main targets of investigations. Thus, differential expression analysis between tumors and adjacent normal tissues is performed prior to prognostic analysis in previous studies. However, recent studies have suggested that the prognostic relevance of differentially expressed genes (DEGs) is insignificant for some cancers, contradicting conventional approaches

**Methods:**

This study investigated the prognostic efficacy of transcriptomic data from tumors and adjacent normal tissues using The Cancer Genome Atlas dataset. Prognostic analysis using Cox regression models and survival prediction using machine‐learning models and feature selection methods were employed.

**Results:**

The results revealed that for kidney, liver, and head and neck cancer, adjacent normal tissues harbored higher proportions of prognostic genes and exhibited better survival prediction performance than tumor tissues and DEGs in machine‐learning models. Furthermore, the application of a distance correlation‐based feature selection method to kidney and liver cancer using external datasets revealed that the selected genes for adjacent normal tissues exhibited higher prediction performance than those for tumor tissues. The study results suggest that the expression levels of genes in adjacent normal tissues are potential prognostic markers. The source code of this study is available at https://github.com/DMCB‐GIST/Survival_Normal.

## Graphical abstract

Tumor adjacent normal tissues of multiple cancer types exhibited higher survival prediction performances using machine learning models. Further, feature selection worked more effectively in tumor adjacent normal tissues. Performances of differentially expressed genes were also compared.

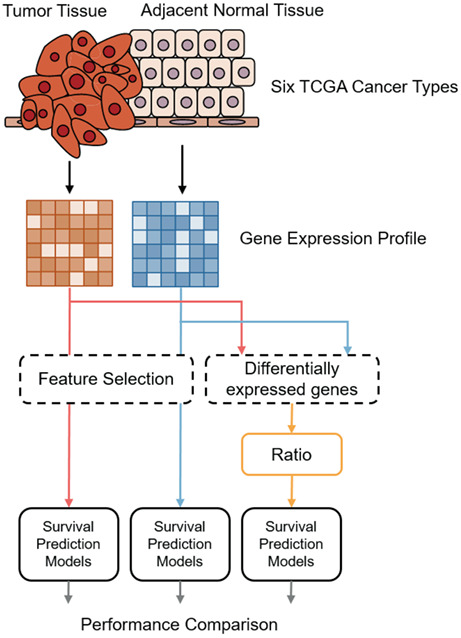

## 1 INTRODUCTION

Cancer prognosis is one of the most important feats of cancer researchers, as a more precise prediction can promote proper clinical treatment for patients. However, cancer prognosis is challenging, as the heterogeneity and environment of different cancer types significantly affect their prognoses.^1^ With the development of sequencing technologies, several genomic and transcriptomic data from cancer patients have been obtained and are publicly available.^2^, ^3^ Therefore, deep learning has made advancements in the biomedical domain, including survival analysis and prediction. Recently, machine learning‐based survival models have been developed and applied to address nonlinear survival data; however, the Cox proportional hazards model is traditionally one standard.^4^, ^5^, ^6^

Transcriptomic data derived from tumor‐adjacent normal tissues are often used as “control” to draw the expression differences of genes between tumor and normal tissues. Conventionally, analysis of gene expression differences has been performed prior to survival analysis, and it resulted in identifying genes that are related to both diagnosis and prognosis. For example, several studies have identified prognostic genes only among differentially expressed gene (DEG) sets.^7^, ^8^, ^9^, ^10^, ^11^ However, the effectiveness of DEGs in survival analyses is understudied.

Most transcriptomic data are generated from tumor tissues, whereas tumor‐adjacent normal tissues have been ill‐studied, making obtaining normal tissue data difficult.^12^ However, recent studies have reported that tumor‐adjacent normal tissues may provide predictive information on patient survival or cancer progression.^12^, ^13^, ^14^ For example, using The Cancer Genome Atlas (TCGA) data, An et al. demonstrated that differential expressions between tumors and adjacent normal tissues were unrelated to the corresponding survival of cancer patients.^15^, ^16^ In addition, several studies have suggested that noncancerous liver tissues improved survival prediction in hepatocellular carcinoma patients, suggesting that tumor‐adjacent normal tissue might hold significant prognostic information.^17^, ^18^, ^19^ Similarly, Zhou et al. studied the relationship between tumor‐adjacent normal tissues and recurrence‐free survival of prostate cancer patients and suggested a potential role for the tumor microenvironment.^20^ However, most previous studies mainly prioritized a single cancer type and did not systemically study multiple cancer types. Also, most studies did not focus on the efficacy of the feature selection steps according to the tumor or adjacent normal tissues.

In this study, the prognostic efficacy of tumor and adjacent normal tissue samples from various cancer patients were investigated, and their performances were compared using transcriptomic and prognostic data from TCGA.^15^ Additionally, the efficacy of the feature selection methods, such as gene expression differences and distance correlation, was compared in a prognostic manner. For screening steps based on distance correlation, kidney and liver cancer datasets were obtained from the International Cancer Genome Consortium (ICGC).^21^ The biological functions of survival‐related gene sets derived from the distance correlation method were explored using enrichment analysis.

## 2 MATERIALS AND METHODS

### 2.1 Study design

**FIGURE 1:**
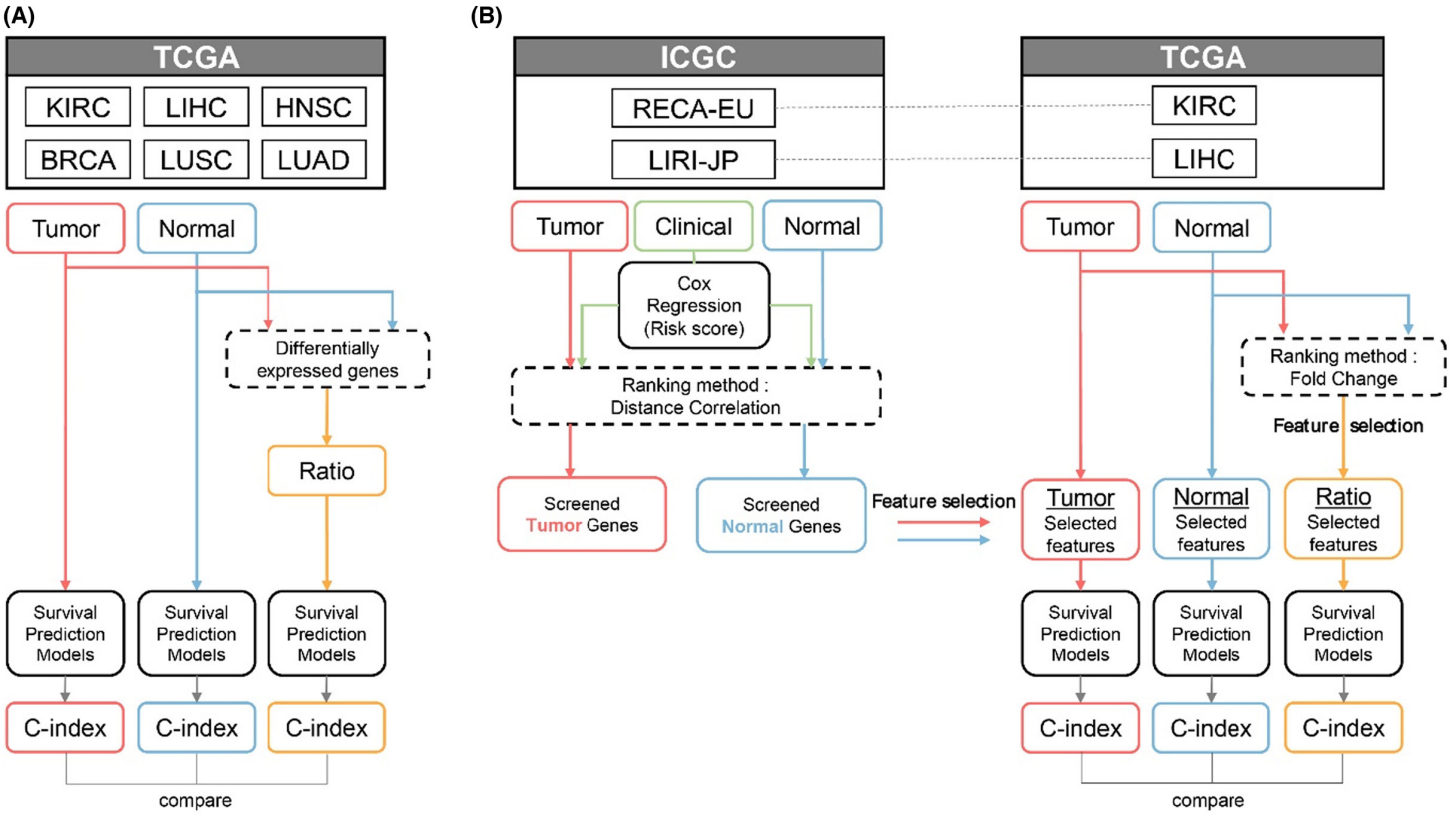
Workflow of the study. (A) The prognostic relevance of transcriptomic profiles of tumor and adjacent normal tissues and gene expression differences were investigated using survival prediction models. (B) Feature selection methods based on distance correlation and fold‐change were applied to kidney and liver cancer datasets to identify prognostic genes and investigate their efficacy in survival predictions.

This study was designed to investigate the prognostic efficacy of transcriptomic profiles of tumors and adjacent normal tissues of cancer patients and analyze the prognostic value of gene expression differences calculated using tumor and normal tissues. Transcriptomic profiles of tumor and adjacent normal tissues and prognostic information of cancer patients were obtained from TCGA and ICGC datasets. Machine learning‐based survival prediction models were employed to compare the prognostic relevance of the transcriptomic profiles of each tissue and their ratio of DEGs (Figure 1A). Model performances were measured using the concordance index (C‐index). Next, feature selection methods were applied for kidney and liver cancer datasets to identify prognostic genes and improve survival prediction performance. For feature selection, distance correlation and fold‐change‐based methods were used for gene ranking, and their prognostic efficacy was investigated based on the performance of the survival prediction models (Figure 1B).

### 2.2 Datasets and preprocessing

TCGA‐Kidney Renal Clear Cell Carcinoma (KIRC), liver hepatocellular carcinoma (LIHC), head and neck squamous cell carcinoma (HNSC), breast cancer (BRCA), lung adenocarcinoma (LUAD), and lung squamous cell carcinoma (LUSC) from the TCGA database were studied as they contained a sufficient number of tumor and paired normal samples and prognostic events. Transcriptome and clinical data were obtained from TCGA Data Portal via the “TCGAbiolinks” package in R.^22^ From the transcriptomic data, Fragments Per Kilobase of transcript per million mapped reads upper quartile (FPKM‐UQ) values of 19,962 protein‐coding genes were used. The FPKM‐UQ method is preferred to obtain a more reliable value than including genes with high variability. Each gene expression matrix was standard normalized using columns (genes). External datasets for kidney cancer (Renal Cell Cancer‐European Union [RECA‐EU]) and liver cancer (Liver Cancer‐RIKEN, Japan [LIRI‐JP]) from the ICGC database were also employed for screening.^21^ They contained RNA‐seq data from the tumor and paired adjacent tissues and prognostic information. The numbers of samples for each data and characteristic are listed in Table 1.

**TABLE 1:** Sample size and data characteristics.

Cancer cohorts		Number of samples	Number of deceased samples	Age in years (median)	Tumor stage			
					I	II	III	IV
TCGA (Paired)	KIRC	72	27 (38%)	38–90 (62)	27	14	29	2
	HNSC	42	32 (76%)	29–87 (64)	3	18	9	12
	LIHC	50	34 (68%)	20–81 (68.5)	20	14	13	3
	BRCA	112	44 (39%)	30–90 (56.5)	29	65	11	7
	LUAD	57	26 (46%)	42–86 (66)	18	36	2	1
	LUSC	49	30 (61%)	45–85 (69)	9	34	5	1
ICGC	RECA‐EU (Normal)	45	17 (38%)	38–83 (62)	26	5	13	1
	RECA‐EU (Tumor)	91	30 (33%)	35–83 (60)	54	13	22	2
	LIRI‐JP (Normal)	202	40 (20%)	31–86 (68)	26	93	65	18
	LIRI‐JP (Tumor)	240	43 (18%)	31–89 (69)	36	109	74	21

In the screening steps, genes with zero mean in the TCGA datasets were discarded, and those that existed in the TCGA and ICGC datasets were retained. For kidney cancer, 16,904 and 17,100 genes were retained for tumor and adjacent normal tissues, respectively, whereas for liver cancer, 11,264 and 11,965 genes were retained, respectively.

### 2.3 Identification of differentially expressed genes and their expression ratio

DESeq2 was used to calculate fold changes and identify DEGs.^23^ Given that only paired tumor and normal data of patients were used, tissue type and patient identifier were used as factor variables in constructing models. A gene was considered a DEG based on an absolute log 2‐fold‐change value of ˃2 and an adjusted *p*‐value of ˂0.05.

DEG expression ratio was calculated for each patient to compare the prognostic value with the expression values of tumor and normal tissues. The expression ratio of the *i*‐th patient of *j*‐th for identifying DEG was calculated using two methods:

(1) $${\text{Ratio}}_{\text{individual}}=\ln\left(\frac{\text{Tumor expressio}n_{\mathrm{ij}}}{\text{Normal expressio}n_{\mathrm{ij}}}\right)$$

(2) $${\text{Ratio}}_{\text{median}}=\ln\left(\frac{\text{Tumor expressio}n_{\mathrm{ij}}}{\text{median}\;\left(\text{Normal expressio}n_{j}\right)}\right)$$

To calculate the log ratio, the normal expression of each patient was used in Equation (1), whereas the median of normal expressions of all patients was used in Equation (2). A comparative analysis between these two approaches was used to investigate the potential survival prediction efficacy of the individuality of normal tissues.

### 2.4 Data screening via distance correlation

Data screening steps were applied to select more reliable survival‐related genes using external datasets from ICGC via distance correlation.^24^, ^25^ Distance correlation measures linear and nonlinear associations between two random variables. The distance correlation between gene expression level and the linear predictor values of Cox regression models using clinical variables, such as patient age and tumor stage, were calculated. Metastasis was only employed for kidney cancer analysis because the LIRI‐JP did not include metastasis information. Genes were ranked according to their distance correlations by tissue type. The “energy” package in R was used for computation.^26^

### 2.5 Survival prediction model and evaluation

Neural networks (NN), random survival forest (RSF), and survival support vector machine (SSVM) were used for cancer prognosis using high‐dimensional RNA‐seq data.^27^ For each data type, a prediction model was trained and tested using 70% of the randomly selected samples and the remainder, respectively. The specific training and test set split were maintained for each model type to control randomness effects. These steps were repeated 50 times to calculate average performances. Model performances were measured using the C‐index, which indicates the proportion of concordant pairs divided by the total number of possible evaluation pairs. The C‐index ranged between 0 and 1, and 0.5, indicating that the performance of a model is equivalent to random guessing.

For RSF and SSVM, the models were fitted with hyper‐parameters searched by threefold cross‐validation. For the RSF models, the number of estimators (n_estimators) and the minimum number of samples required to be at a leaf node (min_samples_leaf) and split an internal node (min_samples_split) were searched. For the SVM models, the kernel type, weight of penalizing the squared hinge loss (alpha), and degree for poly‐kernels (gamma) were searched. Moreover, NN models, including DeepSurv and Cox‐nnet, were designed according to previous studies.^28^, ^29^ The NN models were constructed with two hidden layers, where the dimensions were the square roots of the input dimension. A rectified linear unit (ReLU) and a hyperbolic tangent (Tanh) function were used as activation functions. The models had partial log‐likelihood with regularization as the loss function. With the output of the network $\hat{h_{\theta }}\left(x\right)$, the objective function is set as

(3) $$l\left(\theta \right)=\sum_{i:E\left(i\right)=1}\left(\hat{h_{\theta }}\left(x_{i}\right)-\log\sum_{t_{i}\geq t_{j}}\exp\left(\hat{h_{\theta }}\left(x_{j}\right)\right)\right)+\lambda \left(\theta \right)$$

where $E\left(i\right)$ denotes event occurrence, $t_{i}$ denotes the observed time for the *i*‐th patient, and λ denotes the regularization parameter of the L2 penalty.

### 2.6 Functional annotation

g:Profiler was used to determine the biological roles of survival‐related genes^30^; it offers a functional profiling tool that discovers statistically significantly enriched terms of Gene Ontology (GO) and Kyoto Encyclopedia of Genes and Genomes (KEGG) databases on the input gene list.^31^, ^32^, ^33^, ^34^, ^35^ The top 1000 ranked genes were made based on the distance correlations for each data type as input lists. Biological terms with g:SCS adjusted *p*‐value <0.05 were considered statistically significant.

## 3 RESULTS

### 3.1 Survival analysis with clinical data, gene expression data of tumor and normal tissues, and expression ratio of DEGs

**FIGURE 2:**
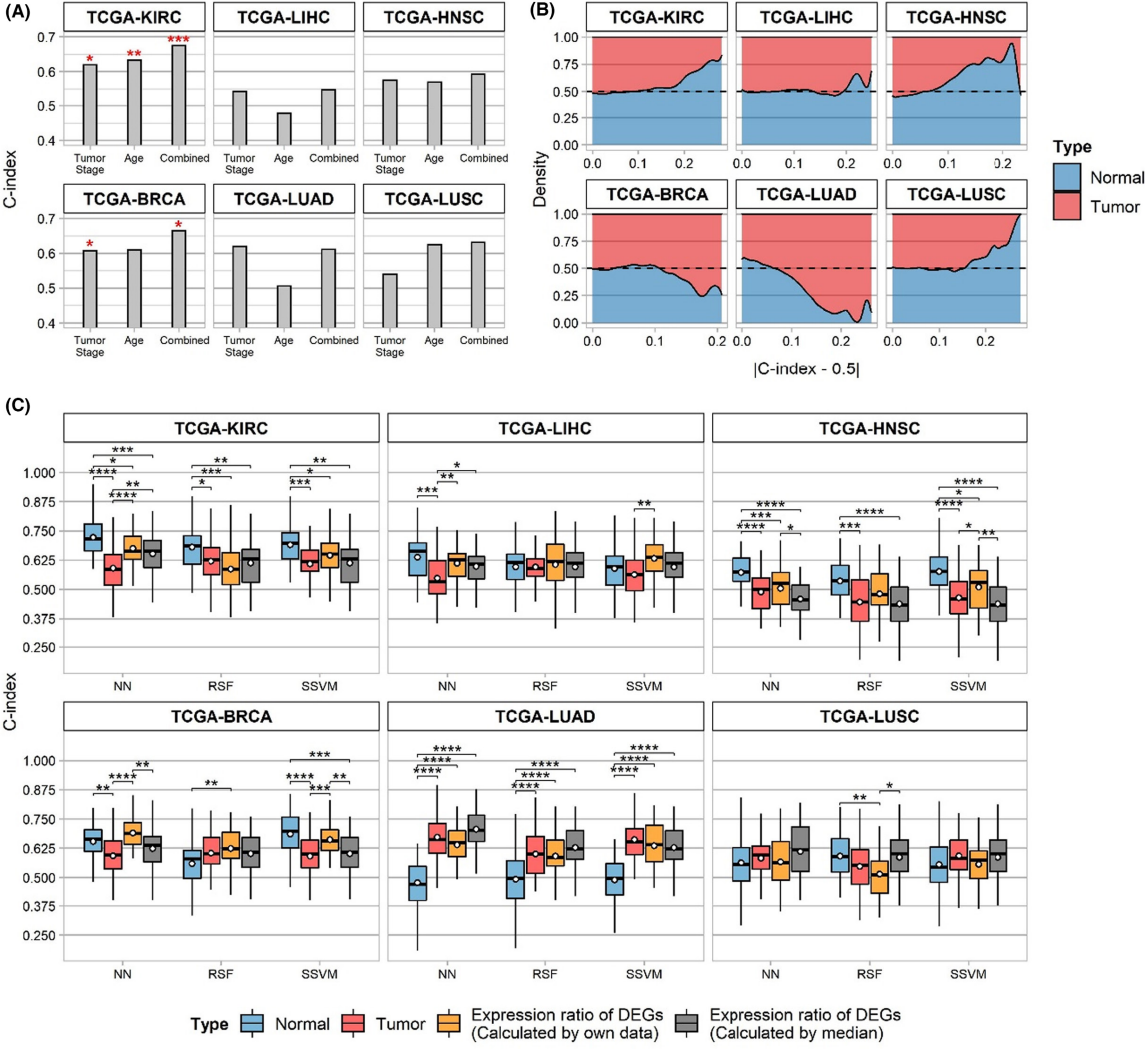
Prognostic information of six The Cancer Genome Atlas (TCGA) datasets. (A) The C‐index of the clinical variables was investigated using the Cox PH model. The significance evaluated by the likelihood‐ratio test is indicated by asterisks above the bars. (B) Stacked density plot of informative genes in a prognostic manner for each tissue type. (C) The survival prediction performance of neural networks (NN), random survival forest (RSF), and survival support vector machine (SSVM), which uses different data types. Yellow boxes indicate the performance ranges using log2 fold‐change of DEGs where the normal tissue expression of the patient was used, whereas gray boxes indicate median normal tissue expression for fold‐change calculation.

Before we examine the prognostic performances of gene expressions, we first examined the relationships between survival and basic clinical variables, including tumor stage and diagnostic age. The Cox regression models were fitted to examine the performance of the clinical variables for each cancer type, as shown in Figure 2A. The prediction performances represented by the C‐index of the fitted Cox regression models showed diverse aspects and difficulties for each cancer type. Statistically, Cox coefficients of tumor stage in the univariate Cox regression models showed *p*‐values of 0.04 and 0.01 by Wald test for TCGA‐KIRC and BRCA, respectively, indicating significant relationships between patient survival. Moreover, patient age showed a significant relationship only for TCGA‐KIRC (*p* = 0.002).

Next, relative proportions of informative genes between tumors and adjacent normal tissues were explored in a prognostic manner (Figure 2B). Given that a C‐index of approximately 0.5 indicates a minute relationship with survival, genes with a C‐index of approximately 0 or 1 were considered informative genes. The C‐index of every gene from the tumor and adjacent normal tissues was investigated, and the number of genes corresponding to specific C‐index values was compared. Adjacent normal tissues of TCGA‐KIRC, LIHC, HNSC, and LUSC had a higher ratio of informative genes than tumor tissues, whereas TCGA‐BRCA and LUAD exhibited contrasting results.

Based on these observations, the prognostic efficacy of gene expression between normal and tumor tissues was compared using three survival prediction models (NN, RSF, and SSVM) for each tissue type; the best model configuration and data types varied according to the cancer type. One of the most crucial findings was that the proportion of informative genes between normal and tumor tissues affected the prediction performance ranges for each cancer type (Figure 2C). For TCGA‐KIRC, LIHC, and HNSC, adjacent normal tissues showed higher average performance than tumor tissues regardless of the prediction model type, consistent with the ratio of informative genes in each tissue. Statistical differences between tissues were validated using the Wilcoxon test, showing adjusted *p*‐values ˂0.05 on NN models. For TCGA‐KIRC and HNSC, significant *p*‐values were observed in the SSVM and RSF models. For KIRC and LIHC, adjacent normal tissues exhibited higher prediction performances than tumor stages and ages combined. Although TCGA‐BRCA tumor tissues showed higher proportions of informative genes, adjacent normal tissues exhibited a better performance range in the NN and SVM models. However, lung cancers had different results compared to other cancer types. For TCGA‐LUAD, tumor tissue had a higher performance than adjacent normal tissue, consistent with the proportions of informative genes. In addition, no significant differences were observed between the tissues for TCGA‐LUSCs.

To investigate the prognostic efficacy of DEGs, the expression ratio of DEGs in machine‐learning models was employed. Two DEG expression ratios were calculated using Equations (1) and (2) in the Method section, which were referred to as “individual ratio” and “median ratio.” The prognostic relevance of DEGs was surmised by the survival prediction results of the NN models (Figure 2C). Although DEGs exhibited better performance than single gene expression data types (normal or tumor tissues) for TCGA‐BRCA, LUAD, and LUSC, they were ineffective for TCGA‐KIRC, LIHC, and HNSC, consistent with the previous results observed. For TCGA‐KIRC and HNSC, adjacent normal tissue exhibited better performance than the expression ratio of DEGs, with a significant *p*‐value (0.011 and $4.84\times 10^{-4}$ for individual ratio and median ratio in KIRC, $7.76\times 10^{-4}$ and $6.06\times 10^{-9}$ in HNSC, respectively). For LIHC, adjacent normal tissue showed optimal performance in NN models, whereas the individual ratio of DEGs was best in SVM models. In contrast, the individual ratio of DEGs in TCGA‐BRCA showed optimal performance in NN models, whereas adjacent normal tissue was best in SVM models.

The prediction performances were compared using the individual and median ratios to investigate the importance of the paired adjacent normal tissue data of a patient. For TCGA‐KIRC, LIHC, HNSC, and BRCA, the individual ratio of DEGs was slightly better than the median ratio. Particularly, the individual ratio of TCGA‐HNSC was significantly higher than the median ratio in the NN and SVM models, as validated by the Wilcoxon test. However, for lung cancer, the individual ratio of DEGs was worse than the median ratio, indicating that paired normal tissue from lung cancer patients was ineffective for survival prediction.

The correlations between log‐fold changes in each gene expression in tumor and normal tissues and their corresponding Cox coefficients were investigated (Figure S1–S3). TCGA‐BRCA and LUAD had Pearson correlation coefficients of 0.153 and 0.207, respectively, whereas TCGA‐LUSC had a correlation coefficient of −0.134. However, TCGA‐KIRC, LIHC, and HNSC showed correlation coefficients of approximately zero, indicating irrelevant correlations.

### 3.2 Prognostic values of selected features for kidney and liver cancer

To select survival‐related gene sets and explore their prognostic efficacies, two feature selection methods were applied: log fold‐change and distance correlation. For the log fold‐change method, the calculation results from DESeq2 were used. Genes were ranked by each method, and the different feature sizes selected from the highest rankings were employed in survival prediction models to investigate feature selection efficacy.

For distance correlation‐based feature screening, RECA‐EU and LIRI‐JP datasets from ICGC, which contain transcriptomic data from tumors and adjacent normal tissues of kidney and liver cancer patients and corresponding prognostic data, were employed. Distance correlation scores were measured between expression levels and predictor variables derived from the Cox regression fitted by clinical variables. The selected genes by ICGC datasets were clustered into favorable or unfavorable genes for survival, as depicted in Figure S2, and they were listed in Table S1.

#### 3.2.1 Efficacy of feature selection

**FIGURE 3:**
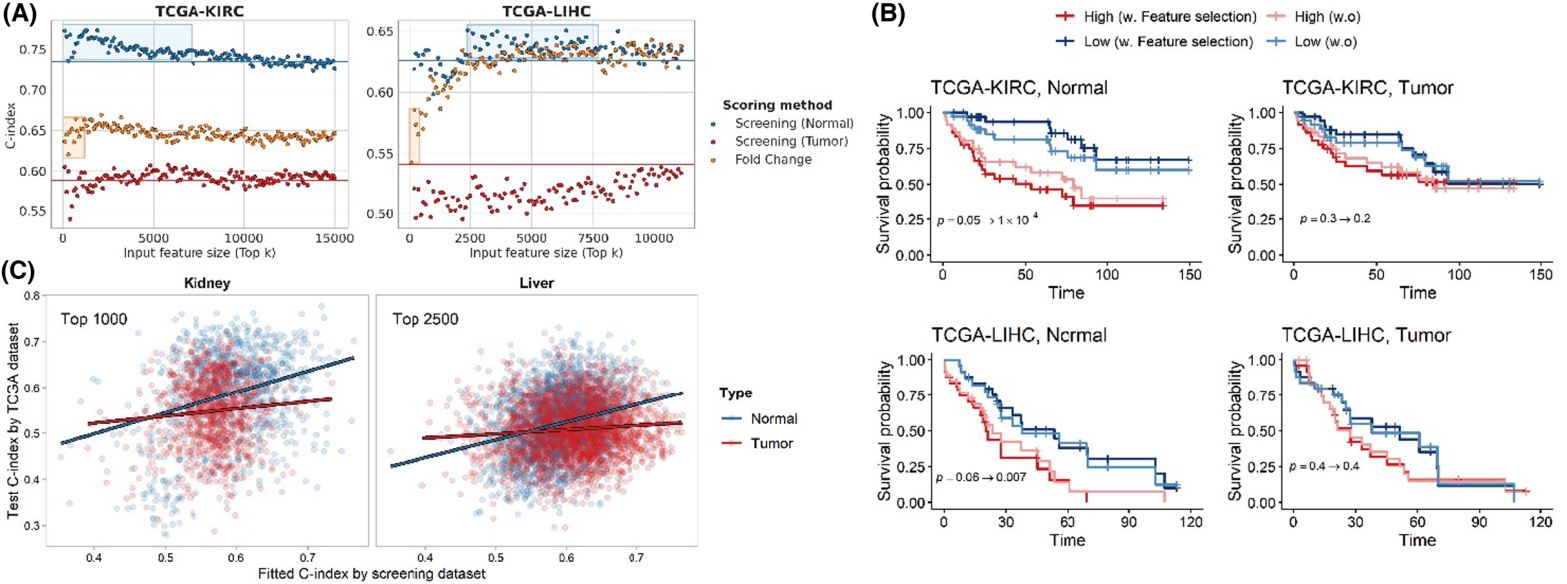
Effectiveness of feature selection for kidney and liver cancer. (A) Survival prediction performance of NN models produced using selected features with different feature sizes. Each dot represents the average performance of 20 iterations. Blue and red solid lines indicate the average performance of adjacent normal and tumor tissues, respectively, without feature selection. Blue boxes show the range in which screening steps exhibited better performance than the baseline for adjacent normal tissues. The yellow boxes include DEGs. (B) Patients are grouped by the median of the NN model output with or without distance correlation‐based screening process, respectively. The screening steps worked more effectively on adjacent normal tissue of kidney and liver cancers. (C) Correlation between fitted C‐index using the screening dataset and test C‐index using TCGA dataset by univariate Cox PH models.

The NN model training and testing were performed with different input sizes of selected features in sorted order for each method. Adjacent normal tissues exhibited higher performance ranges than tumor tissues, regardless of different feature sizes and prediction models, confirming previous observations (Figure 3A and Figure S2). The optimal performances of adjacent normal tissues in kidney and liver cancers were 0.773 and 0.651, respectively; they showed improvements compared to those without the feature selection steps. In contrast, log fold changes of DEGs showed intermediate performance ranges between tumor and adjacent normal tissues for kidney and liver cancers in NN models. The selected features in adjacent normal tissues also exhibited better performance ranges in the SVM and RSF models, whereas tumor tissues did not (Figure S3).

To determine the effectiveness of feature selection based on distance correlation, patients were grouped into high or low risk group by median of output values of the NN models with or without the feature selection step, respectively. Input feature sizes were determined based on the observations with the highest performances. For KIRC, input feature sizes were 1300 and 5800 for normal and tumor, respectively, and for LIHC, those were 2300 and 10,200, respectively. As illustrated in Figure 3B, the feature selection process made patients grouped clearer in most cases. Especially, feature selection worked more effectively for adjacent normal tissues. The *p*‐values of the Kaplan–Meier estimators decreased less than 0.05 after the feature selection steps were applied. Specifically, *p*‐values were dropped to 0.05 to 0.0001 for KIRC and 0.06 to 0.007 for LIHC. However, the results with tumor tissues did not exhibit the noteworthy difference after feature selection. For KIRC tumor data, *p*‐value was dropped 0.3 to 0.2 while LIHC tumor data remained the same as 0.4, which were not significant in both cases.

#### 3.2.2 Screening steps were more effective for adjacent normal tissues than tumor tissues

For adjacent normal tissues, patients were more clearly separated with screening steps. Also, specific ranges of feature sizes were observed where the performance was better than without it. However, screening steps were ineffective for tumor tissues. Therefore, it was hypothesized that tumor tissue heterogeneity between TCGA and ICGC datasets might influence feature selection during the screening steps. Univariate Cox regression models were fitted using expression values of each gene in the ICGC dataset, and using the corresponding gene in the TCGA dataset, the C‐index was tested using the Cox regression models. Pearson correlation coefficients were measured between the training C‐index and test C‐index. Adjacent normal tissues from kidney and liver cancer datasets exhibited higher similarities (0.293 and 0.262, respectively) between TCGA and ICGC datasets than tumor tissues, which showed almost no correlation (0.085 and 0.091, respectively) (Figure 3C). These differences in similarities explain that the better efficacy of feature selection methods on adjacent normal tissues using external datasets is anticipated.

### 3.3 Functional annotation of survival‐related genes

**FIGURE 4:**
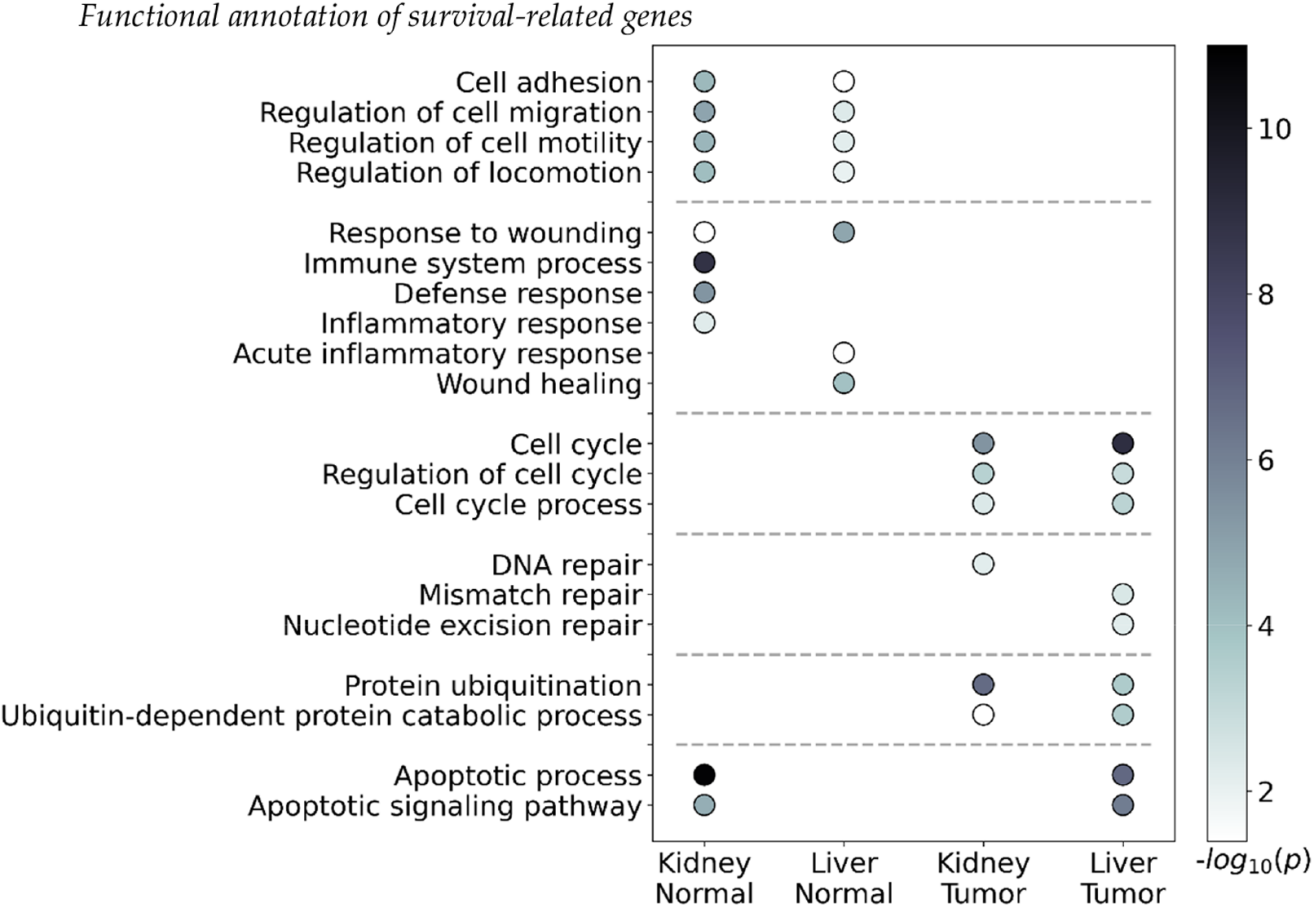
Functional annotation of top 1000 screening genes based on distance correlations.

Biological functions of the top 1000 screened genes based on distance correlations for each category were investigated based on GO and KEGG databases, and notable terms are presented in Figure 4. The results revealed that cell movement and immune‐related pathways were enriched by normal tissue gene sets from the kidney and liver; relationships between tumor microenvironments and cell movements have been reported in several previous studies, along with immune responses.^36^, ^37^ However, cell cycle, DNA repair, and protein ubiquitination‐related pathways were enriched by tumor tissue gene sets; the associations between these pathways and cancers are well‐known.^38^, ^39^, ^40^ Apoptosis‐related pathways were enriched by the normal kidney and liver tumor gene sets.

In addition to the terms depicted in Figure 4, metabolic and catabolic process‐related pathways were frequently enriched by all gene sets. Lipid and sugar metabolic process‐related pathways were enriched by normal liver tissue gene sets.

## 4 DISCUSSION

Recently, the effects of tumor microenvironment on cancer progression and prognosis have been investigated in various aspects including the immune system or crosstalk between tumor tissues.^41^, ^42^, ^43^ In addition, diagnostic or prognostic markers have been discovered from tumor‐adjacent normal tissues. For example, aging‐related genes identified from normal kidney tissues were validated in vivo by cancer invasion.^44^ Despite these interests in tumor microenvironment, few cancer patients produce transcriptomic profiles from tumor‐adjacent normal tissues.

In this study, we collected individual adjacent normal tissues from the TCGA database and investigated the potential patient survival prediction efficacy. Initially, we utilized gene expression data of protein‐coding genes without selecting features to verify whether adjacent normal tissues worthy for prognostic analysis. For TCGA‐KIRC, LIHC, and HNSC, gene expression data from adjacent normal tissues exhibited better survival prediction performance than tumor tissues across multiple types of machine‐learning models, suggesting that adjacent normal tissues might harbor predictive information. In contrast, for lung cancers, adjacent normal tissues exhibited a worse performance than tumor tissues, implying that simple utilization of normal lung tissue data could interrupt survival prediction.

Gene expressions of adjacent normal tissues were usually employed for calculating expression differences between expression levels of corresponding tumor tissues or producing immune infiltration levels.^45^, ^46^, ^47^ Instead of these previous applications, we adopted the distance correlation method to identify prognostic genes. For kidney and liver cancer, screening datasets (RECA‐EU and LIRI‐JP, respectively) were employed. Feature selection by the distance correlation method exhibited performance improvements only in adjacent normal tissues. Across TCGA and screening datasets, adjacent normal tissue data had higher similarities than tumor tissue data in prognosis, suggesting that they share more common features than tumor tissues, which resulted in the efficacy of feature selection. We suppose that heterogeneity across normal tissues was less than that of tumor tissues, making feature selection effective.

We underwent the literature search for top ranked genes in normal tissues. For kidney cancer, it was reported that the expression of CD74 is increased in diseased kidney and promotes an inflammatory response and may protect from interstitial kidney fibrosis.^48^, ^49^ GRAMD1A is related to immune infiltration in tumor microenvironments, and expression of GRAMD1A is significantly related to the survival of kidney cancer patients.^50^ In fact, GRAMD family expression was associated with the survival outcome of the KIRC cohort.^51^ Next, the expression of CYP3A in normal kidney tissue might be involved in tumor development.^52^ KRT8 overexpression in renal cancer was associated with cell migration and invasion, and was significantly related to poor survival of patients.^53^ For liver cancer, suppression of HPD promotes tumorigenesis and cell proliferation.^54^ P4HA1 expression was correlated with infiltration levels of immunosuppressive cells in most cancer types.^55^ High ARID3C expression reduced the survival of hepatocellular carcinoma patients, and the ARID family of genes are known to contribute to the development of tumors.^56^

Selected prognostic genes discovered from adjacent normal tissues were frequently related to cell motility. Several studies discovered that the motility of both tumor and normal cells affects to tumor metastasis in several ways, such as invading of basement membrane, escape from the primary tumor of origin, migration to blood and lymphatic vessels.^57^, ^58^ We suppose that the motility of cells near tumor tissues might cause metastasis, reducing the chance of survival.

Given that transcriptomic data from adjacent normal tissues and prognostic information are rare, obtaining various types and large amounts of data was difficult. This study was limited by the scarcity of these data. Given that an external dataset for screening only kidney and liver cancer was obtained, the distance correlation method could not be applied to other cancer types. In addition, the survival prediction performance of machine‐learning models fluctuated significantly owing to the small sample sizes, making the observation of stable performance scores difficult.

For future work, developing a novel way to combine both paired tumor and normal tissues is possible. As this present work showed, expression differences may not be the best way for identifying prognostic genes for multiple types of cancers.

## 5 CONCLUSIONS

In this study, the prognostic efficacy of transcriptomic data of tumors and adjacent normal samples from TCGA datasets were investigated. Higher proportions of informative genes were observed in adjacent normal tissues than in tumor tissues for kidney, liver, and head and neck cancer. In addition, adjacent normal tissues for these cancers exhibited higher survival prediction performance than tumor tissues in various machine‐learning models. These results suggest that adjacent normal tissues hold more predictive information on the survival of cancer patients and can be potentially used as prognostic markers. The prognostic efficacy of DEGs, which were considered the starting point for identifying prognostic genes in most previous studies, was investigated. Although DEGs exhibited good survival prediction in some cancer types, such as breast and lung cancers, they were ineffective for kidney, liver, and head and neck cancers, suggesting that analyzing differential expression between tumors and adjacent normal tissues may not always be the best method for identifying prognostic genes.

## AUTHOR CONTRIBUTIONS

**Euiyoung Oh:** Data curation (equal); methodology (equal); software (equal); writing – original draft (equal); writing – review and editing (equal). **Hyunju Lee:** Data curation (equal); funding acquisition (equal); investigation (equal); project administration (equal); supervision (equal); writing – original draft (equal); writing – review and editing (equal).

## FUNDING INFORMATION

This work was funded by the Institute of Information & Communications Technology Planning & Evaluation (IITP) grant funded by the Korean government (MSIT) [No. 2019–0‐00567, Development of Intelligent SW systems for uncovering genetic variation and developing personalized medicine for cancer patients with unknown molecular genetic mechanisms, No. 2019–0‐01842, Artificial Intelligence Graduate School Program (GIST)] and GIST Research Project grant funded by the GIST in 2023.

## CONFLICT OF INTEREST STATEMENT

The authors declare no conflict of interest.

## INSTITUTIONAL REVIEW BOARD STATEMENT

Not applicable.

## INFORMED CONSENT STATEMENT

Not applicable.

## Supporting information


Figure S1–S3:

Table S1:

## Data Availability

RNA‐sequencing data and clinical information are available in the National Cancer Institute GDC Data Portal (https://portal.gdc.cancer.gov/).

## References

[1] Liñares‐Blanco J , Pazos A , Fernandez‐Lozano C . Machine learning analysis of TCGA cancer data. PeerJ Comput Sci. 2021;7:e584. doi:10.7717/peerj-cs.584 PMC8293929 34322589

[2] Peng L , Bian XW , Li DK , et al. Large‐scale RNA‐seq transcriptome analysis of 4043 cancers and 548 normal tissue controls across 12 TCGA cancer types. Sci Rep. 2015;5:13413. doi:10.1038/srep13413 26292924 PMC4544034

[3] Tran KA , Kondrashova O , Bradley A , Williams ED , Pearson JV , Waddell N . Deep learning in cancer diagnosis, prognosis and treatment selection. Genome Med. 2021;13:152. doi:10.1186/s13073-021-00968-x 34579788 PMC8477474

[4] Kaur I , Doja MN , Ahmad T . Data mining and machine learning in cancer survival research: an overview and future recommendations. J Biomed Inform. 2022;128:104026. doi:10.1016/j.jbi.2022.104026 35167976

[5] Zhu W , Xie L , Han J , Guo X . The application of deep learning in cancer prognosis prediction. Cancer. 2020;12:603. 10.3390/cancers12030603 PMC7139576 32150991

[6] Cox DR . Regression models and life‐tables. J R Stat Soc B Methodol. 1972;34:187‐220.

[7] Zheng Y , Liu Y , Zhao S , et al. Large‐scale analysis reveals a novel risk score to predict overall survival in hepatocellular carcinoma. Cancer Manag Res. 2018;10:6079‐6096. doi:10.2147/cmar.S181396 30538557 PMC6252784

[8] Zhang W , Shen Y , Feng G . Predicting the survival of patients with lung adenocarcinoma using a four‐gene prognosis risk model. Oncol Lett. 2019;18:535‐544. doi:10.3892/ol.2019.10366 31289525 PMC6539490

[9] Chen Z , Liu G , Hossain A , et al. A co‐expression network for differentially expressed genes in bladder cancer and a risk score model for predicting survival. Hereditas. 2019;156:24. doi:10.1186/s41065-019-0100-1 31333338 PMC6617625

[10] Zhao J , Guo C , Ma Z , Liu H , Yang C , Li S . Identification of a novel gene expression signature associated with overall survival in patients with lung adenocarcinoma: a comprehensive analysis based on TCGA and GEO databases. Lung Cancer. 2020;149:90‐96. doi:10.1016/j.lungcan.2020.09.014 33002836

[11] Li Y , Gu J , Xu F , Zhu Q , Ge D , Lu C . Transcriptomic and functional network features of lung squamous cell carcinoma through integrative analysis of GEO and TCGA data. Sci Rep. 2018;8:15834. doi:10.1038/s41598-018-34160-w 30367091 PMC6203807

[12] Huang X , Stern DF , Zhao H . Transcriptional profiles from paired Normal samples offer complementary information on cancer patient survival–evidence from TCGA pan‐cancer data. Sci Rep. 2016;6:20567. doi:10.1038/srep20567 26837275 PMC4738355

[13] Hu S , Yuan H , Li Z , et al. Transcriptional response profiles of paired tumor‐normal samples offer novel perspectives in pan‐cancer analysis. Oncotarget. 2017;8:41334‐41347. doi:10.18632/oncotarget.17295 28489584 PMC5522216

[14] Frost HR . Analyzing cancer gene expression data through the lens of normal tissue‐specificity. PLoS Comput Biol. 2021;17:e1009085. doi:10.1371/journal.pcbi.1009085 34143767 PMC8244857

[15] Chang K , Creighton CJ , Davis C , et al. The cancer genome atlas pan‐cancer analysis project. Nat Genet. 2013;45:1113‐1120. doi:10.1038/ng.2764 24071849 PMC3919969

[16] An N , Yu Z , Yang X . Expression differentiation is not helpful in identifying prognostic genes based on TCGA datasets. Mol Ther‐Nucleic Acids. 2018;11:292‐299. 29858064 10.1016/j.omtn.2018.02.013 PMC5992444

[17] Hao K , Lamb J , Zhang C , et al. Clinicopathologic and gene expression parameters predict liver cancer prognosis. BMC Cancer. 2011;11:481. doi:10.1186/1471-2407-11-481 22070665 PMC3240666

[18] Hoshida Y , Villanueva A , Kobayashi M , et al. Gene expression in fixed tissues and outcome in hepatocellular carcinoma. N Engl J Med. 2008;359:1995‐2004. doi:10.1056/NEJMoa0804525 18923165 PMC2963075

[19] Budhu A , Forgues M , Ye Q‐H , et al. Prediction of venous metastases, recurrence, and prognosis in hepatocellular carcinoma based on a unique immune response signature of the liver microenvironment. Cancer Cell. 2006;10:99‐111. doi:10.1016/j.ccr.2006.06.016 16904609

[20] Zhou R , Feng Y , Ye J , et al. Prediction of biochemical recurrence‐free survival of prostate cancer patients leveraging multiple gene expression profiles in tumor microenvironment. Front Oncol. 2021;11:632571. doi:10.3389/fonc.2021.632571 34631510 PMC8495167

[21] Hudson TJ , Anderson W , Artez A , et al. International network of cancer genome projects. Nature. 2010;464:993‐998. doi:10.1038/nature08987 20393554 PMC2902243

[22] Colaprico A , Silva TC , Olsen C , et al. TCGAbiolinks: an R/Bioconductor package for integrative analysis of TCGA data. Nucleic Acids Res. 2016;44:e71. 26704973 10.1093/nar/gkv1507 PMC4856967

[23] Love MI , Huber W , Anders S . Moderated estimation of fold change and dispersion for RNA‐seq data with DESeq2. Genome Biol. 2014;15:550. doi:10.1186/s13059-014-0550-8 25516281 PMC4302049

[24] Zhu Z , Fan Y , Kong Y , Lv J , Sun F . DeepLINK: deep learning inference using knockoffs with applications to genomics. Proc Natl Acad Sci. 2021;118:e2104683118. 34480002 10.1073/pnas.2104683118 PMC8433583

[25] Gao L , Fan Y , Lv J , Shao Q‐M . Asymptotic distributions of high‐dimensional distance correlation inference. Ann Stat. 2021;49:1999‐2020. 34621096 10.1214/20-aos2024 PMC8491772

[26] Rizzo ML , Szekely GJ . Energy: E‐statistics: multivariate inference via the energy of data (R package), Version 1.7‐0. 2017.

[27] Pölsterl S . Scikit‐survival: a library for time‐to‐event analysis built on top of scikit‐learn. J Mach Learn Res. 2020;21:1‐6.34305477

[28] Katzman JL , Shaham U , Cloninger A , Bates J , Jiang T , Kluger Y . DeepSurv: personalized treatment recommender system using a Cox proportional hazards deep neural network. BMC Med Res Methodol. 2018;18:24. doi:10.1186/s12874-018-0482-1 29482517 PMC5828433

[29] Ching T , Zhu X , Garmire LX . Cox‐nnet: an artificial neural network method for prognosis prediction of high‐throughput omics data. PLoS Comput Biol. 2018;14:e1006076. 29634719 10.1371/journal.pcbi.1006076 PMC5909924

[30] Raudvere U , Kolberg L , Kuzmin I , et al. G: profiler: a web server for functional enrichment analysis and conversions of gene lists (2019 update). Nucleic Acids Res. 2019;47:W191‐W198. 31066453 10.1093/nar/gkz369 PMC6602461

[31] Ashburner M , Ball CA , Blake JA , et al. Gene ontology: tool for the unification of biology. The Gene Ontology Consortium. Nat Genet. 2000;25:25‐29. doi:10.1038/75556 10802651 PMC3037419

[32] Gene Ontology Consortium . The Gene Ontology resource: enriching a GOld mine. Nucleic Acids Res. 2021;49:D325‐D334. 33290552 10.1093/nar/gkaa1113 PMC7779012

[33] Kanehisa M , Goto S . KEGG: Kyoto encyclopedia of genes and genomes. Nucleic Acids Res. 2000;28:27‐30. doi:10.1093/nar/28.1.27 10592173 PMC102409

[34] Kanehisa M . Toward understanding the origin and evolution of cellular organisms. Protein Sci. 2019;28:1947‐1951. doi:10.1002/pro.3715 31441146 PMC6798127

[35] Kanehisa M , Furumichi M , Sato Y , Ishiguro‐Watanabe M , Tanabe M . KEGG: integrating viruses and cellular organisms. Nucleic Acids Res. 2021;49:D545‐D551. 33125081 10.1093/nar/gkaa970 PMC7779016

[36] Varn FS , Wang Y , Mullins DW , Fiering S , Cheng C . Systematic pan‐cancer analysis reveals immune cell interactions in the tumor Microenvironment Pan‐cancer analysis of immune cell interactions. Cancer Res. 2017;77:1271‐1282. 28126714 10.1158/0008-5472.CAN-16-2490 PMC5798883

[37] Friedl P , Alexander S . Cancer invasion and the microenvironment: plasticity and reciprocity. Cell. 2011;147:992‐1009. doi:10.1016/j.cell.2011.11.016 22118458

[38] Friedberg EC , Aguilera A , Gellert M , et al. DNA repair: from molecular mechanism to human disease. DNA Repair. 2006;5:986‐996. 16955546 10.1016/j.dnarep.2006.05.005

[39] Sun T , Liu Z , Yang Q . The role of ubiquitination and deubiquitination in cancer metabolism. Mol Cancer. 2020;19:1‐19. 33004065 10.1186/s12943-020-01262-x PMC7529510

[40] Matthews HK , Bertoli C , de Bruin RA . Cell cycle control in cancer. Nat Rev Mol Cell Biol. 2022;23:74‐88. 34508254 10.1038/s41580-021-00404-3

[41] Soysal SD , Tzankov A , Muenst SE . Role of the tumor microenvironment in breast cancer. Pathobiology. 2015;82:142‐152. doi:10.1159/000430499 26330355

[42] Wu J , Li L , Zhang H , et al. A risk model developed based on tumor microenvironment predicts overall survival and associates with tumor immunity of patients with lung adenocarcinoma. Oncogene. 2021;40:4413‐4424. doi:10.1038/s41388-021-01853-y 34108619

[43] Whiteside TL . The tumor microenvironment and its role in promoting tumor growth. Oncogene. 2008;27:5904‐5912. doi:10.1038/onc.2008.271 18836471 PMC3689267

[44] Oh E , Kim J‐H , Um J , Jung D‐W , Williams DR , Lee H . Genome‐wide transcriptomic analysis of non‐tumorigenic tissues reveals aging‐related prognostic markers and drug targets in renal cell carcinoma. Cancers (Basel). 2021;13:3045. 34207247 10.3390/cancers13123045 PMC8234889

[45] Qin R , Peng W , Wang X , et al. Identification of genes related to immune infiltration in the tumor microenvironment of cutaneous melanoma. Front Oncol. 2021;11:615963. doi:10.3389/fonc.2021.615963 34136377 PMC8202075

[46] Xiong Y , Wang K , Zhou H , Peng L , You W , Fu Z . Profiles of immune infiltration in colorectal cancer and their clinical significant: a gene expression‐based study. Cancer Med. 2018;7:4496‐4508. doi:10.1002/cam4.1745 30117315 PMC6144159

[47] Deng L , Lu D , Bai Y , Wang Y , Bu H , Zheng H . Immune profiles of tumor microenvironment and clinical prognosis among women with triple‐negative breast cancer. Cancer Epidemiol Biomarkers Prev. 2019;28:1977‐1985. doi:10.1158/1055-9965.Epi-19-0469 31533938

[48] Valiño‐Rivas L , Baeza‐Bermejillo C , Gonzalez‐Lafuente L , Sanz AB , Ortiz A , Sanchez‐Niño MD . CD74 in kidney disease. Front Immunol. 2015;6:483. doi:10.3389/fimmu.2015.00483 26441987 PMC4585214

[49] Sanchez‐Niño MD , Sanz AB , Ruiz‐Andres O , et al. MIF, CD74 and other partners in kidney disease: tales of a promiscuous couple. Cytokine Growth Factor Rev. 2013;24:23‐40. doi:10.1016/j.cytogfr.2012.08.001 22959722

[50] Liu Y , Fu S , Zhang Z , et al. GRAMD1A is a biomarker of kidney renal clear cell carcinoma and is associated with immune infiltration in the tumour microenvironment. Dis Markers. 2022;2022:5939021‐5939025. doi:10.1155/2022/5939021 35860689 PMC9293538

[51] Ng MYW , Charsou C , Lapao A , et al. The cholesterol transport protein GRAMD1C regulates autophagy initiation and mitochondrial bioenergetics. Nat Commun. 2022;13:6283. doi:10.1038/s41467-022-33933-2 36270994 PMC9586981

[52] Murray GI , McFadyen MC , Mitchell RT , Cheung YL , Kerr AC , Melvin WT . Cytochrome P450 CYP3A in human renal cell cancer. Br J Cancer. 1999;79:1836‐1842. doi:10.1038/sj.bjc.6690292 10206301 PMC2362772

[53] Tan HS , Jiang WH , He Y , et al. KRT8 upregulation promotes tumor metastasis and is predictive of a poor prognosis in clear cell renal cell carcinoma. Oncotarget. 2017;8:76189‐76203. doi:10.18632/oncotarget.19198 29100303 PMC5652697

[54] Tong M , Wong T‐L , Zhao H , et al. Loss of tyrosine catabolic enzyme HPD promotes glutamine anaplerosis through mTOR signaling in liver cancer. Cell Rep. 2021;36:109617. doi:10.1016/j.celrep.2021.109617 34433044

[55] Zhao Q , Liu J . P4HA1, a prognostic biomarker that correlates with immune infiltrates in lung adenocarcinoma and pan‐cancer. Front Cell Dev Biol. 2021;9:754580. doi:10.3389/fcell.2021.754580 34966739 PMC8710955

[56] Sun J , Cheng N‐S . Comprehensive landscape of ARID family members and their association with prognosis and tumor microenvironment in hepatocellular carcinoma. J Immunol Res. 2022;2022:1688460. doi:10.1155/2022/1688460 35402625 PMC8986425

[57] Stuelten CH , Parent CA , Montell DJ . Cell motility in cancer invasion and metastasis: insights from simple model organisms. Nat Rev Cancer. 2018;18:296‐312. doi:10.1038/nrc.2018.15 29546880 PMC6790333

[58] Xu L , Gordon R , Farmer R , et al. Precision therapeutic targeting of human cancer cell motility. Nat Commun. 2018;9:2454. doi:10.1038/s41467-018-04465-5 29934502 PMC6014988

