# Supplementary material for: Transcriptomic data in tumor‐adjacent normal tissues harbor prognostic information on multiple cancer types
Source: Cancer Med. 2023 Mar 31;12(10):11960–70. doi: 10.1002/cam4.5864 (PMC10242347; doi:10.1002/cam4.5864)
Supplement: Supplementary file 1 — Figure S1–S3: [file CAM4-12-11960-s002.pdf]

Supplemental Figures

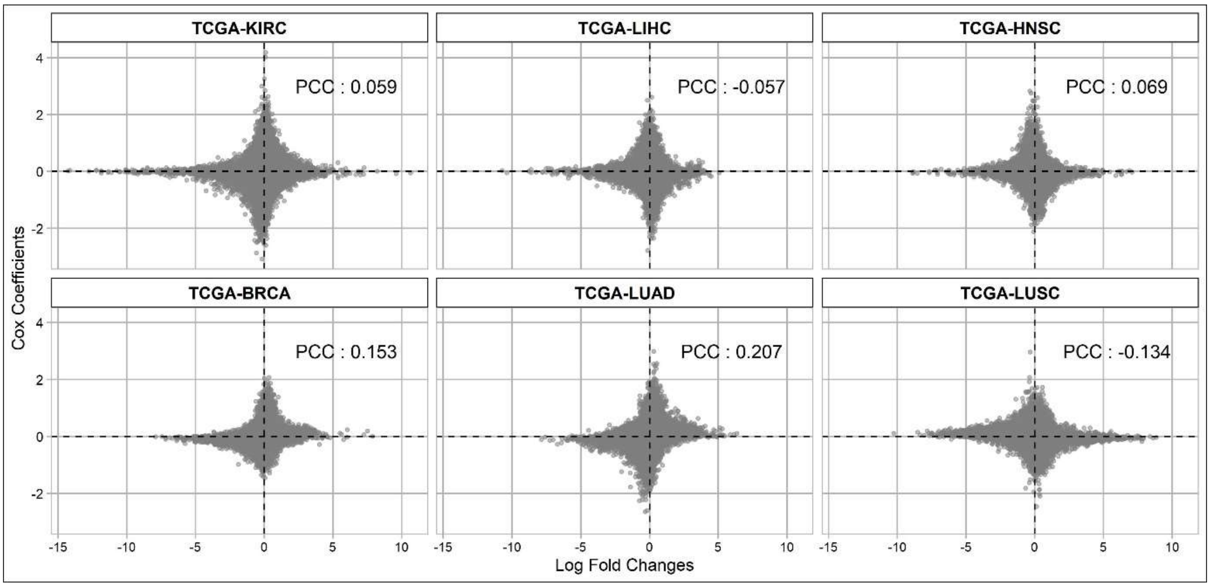

Figure S1. Relationships between log fold change and Cox coefficients. Scatter plots of log fold changes and Cox coefficients were fitted by the expression ratio of each gene. The Pearson correlation coefficients between them are shown.

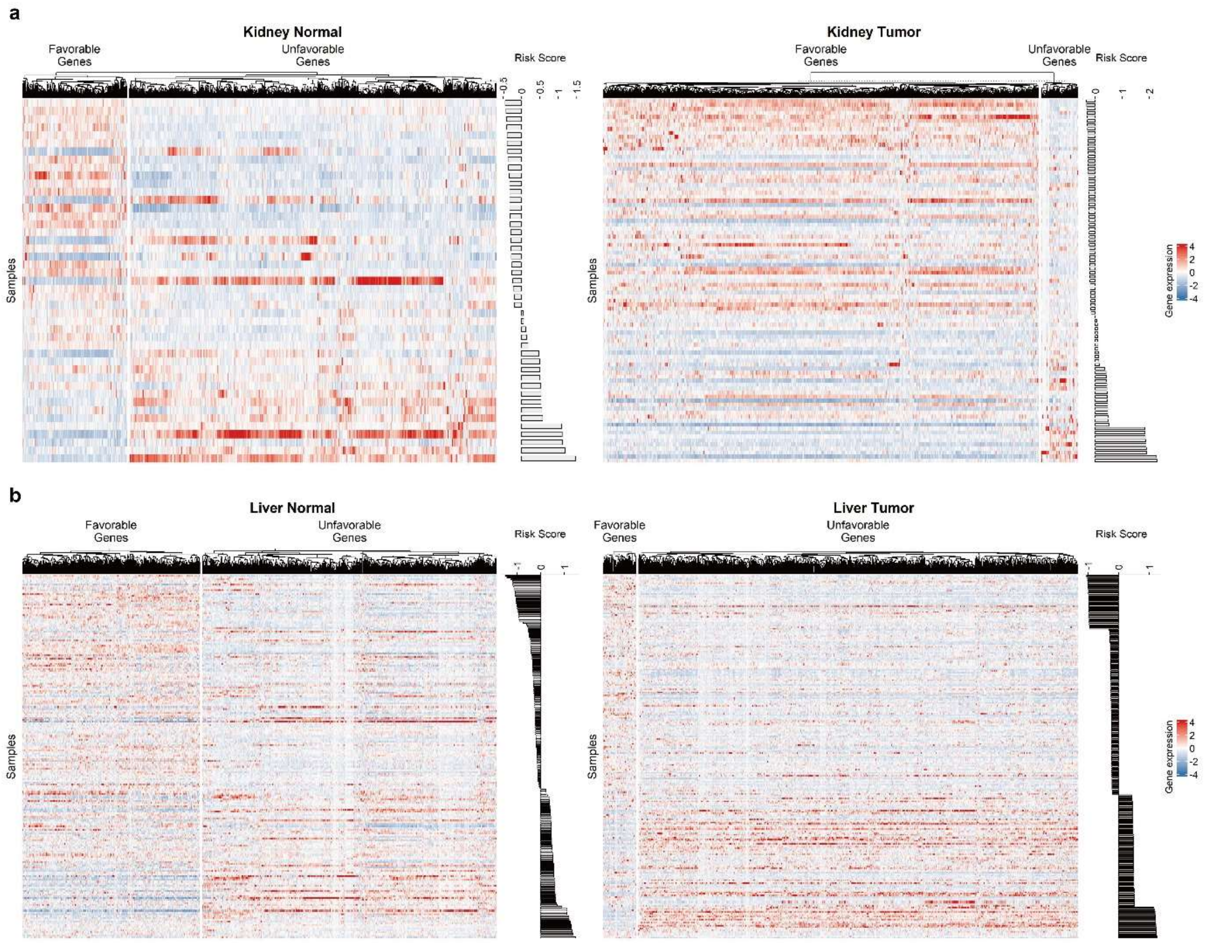

Figure S2. Heatmap of ICGC dataset with top 1000 screening genes. Patients were sorted by their risk scores, which were derived by Cox regression employing clinical variables (tumor stage and age). Top ranked genes were clustered into favorable or unfavorable genes with survival.

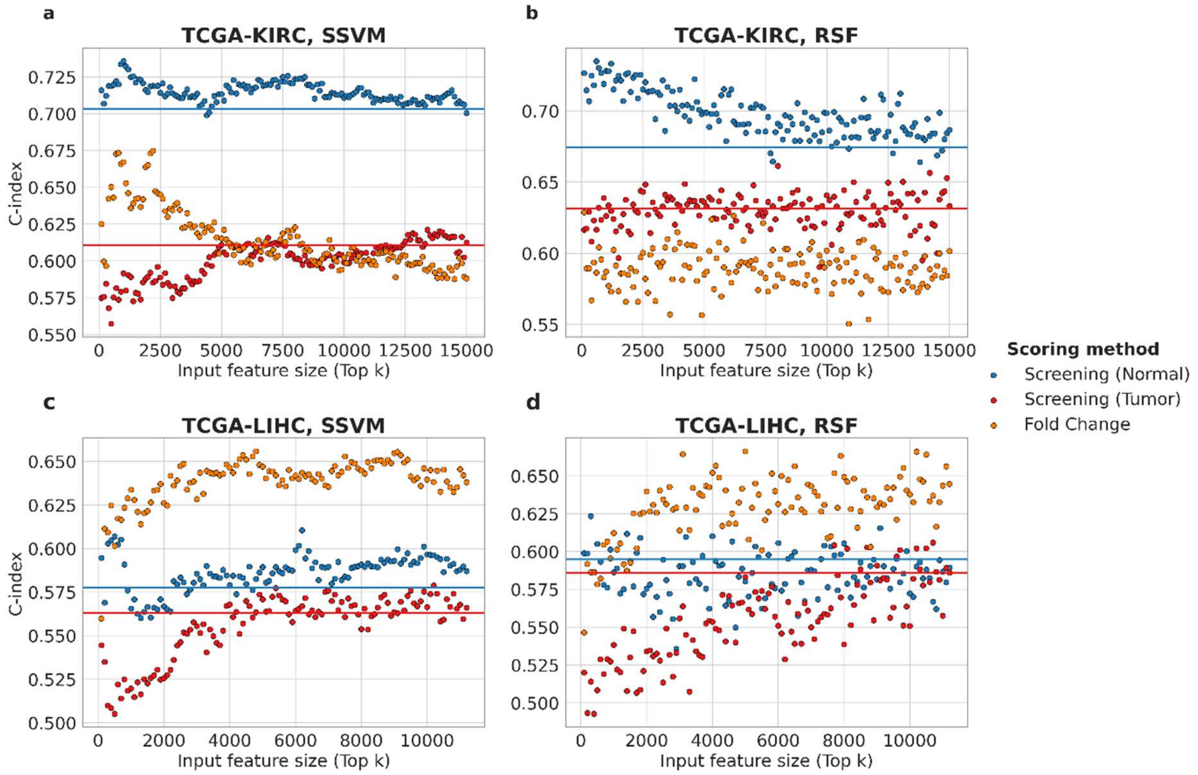

Figure S3. Survival prediction performances of SVM and RF models with feature selection methods. Each dot represents the average performance of 20 iterations. The solid lines indicate the average performances with no feature selection for each model type. Regardless of the model type and feature sizes, adjacent normal tissues exhibited better performance ranges than tumor tissues.
