# Supplementary material for: Transcriptomic data in tumor‐adjacent normal tissues harbor prognostic information on multiple cancer types
Source: Cancer Med. 2023 Mar 31;12(10):11960–70. doi: 10.1002/cam4.5864 (PMC10242347; doi:10.1002/cam4.5864)
Supplement: Supplementary file 2 — Table S1: [file CAM4-12-11960-s001.pdf]

Table S1; Screening gene list by ICGC datasets.

Rank	Kideny Normal	Kidney Tumor	Liver Normal	Liver Tumor	Rank	Kidney Normal	Kidney Tumor	Liver Normal	Liver Tumor
**1**	CD74	SIRPG	HPD	MRPS24	**501**	BDKRB2	PHLDB2	ASAP1	CHCHD5
**2**	NKAIN1	ASIC1	P4HA1	URGCP-MRPS24	**502**	PLEKHA5	CNOT8	SLC23A1	NARF
**3**	GRAMD1A	TNS4	AGMAT	FUNDC2	**503**	PHETA1	ALS2	SFXN5	UBE2J2
**4**	CYP3A4	CCN1	ARID3C	LPCAT1	**504**	LRRC42	APLF	TMOD3	ZCRB1
**5**	KRT8	RTP3	ANGPT2	EIF3B	**505**	WDR45	MSR1	NME1-NME2	TIMMDC1
**6**	STAP2	DCXR	DNAJC16	EXOC4	**506**	CSK	SLC40A1	ID2	RPL13
**7**	OTOS	ECEL1	GLYAT	ENO1	**507**	ALYREF	PNRC2	SLC39A5	C1R
**8**	IPO4	ZNF100	DLL4	EIF4A3	**508**	MYL9	USP34	CPD	RPS6
**9**	MME	IQCA1	PIPOX	MFSD5	**509**	TTLL1	TANK	PLAU	ACVR1
**10**	SLC35F6	HAUS3	EPHX1	ARPC1A	**510**	NT5DC2	EPC2	ESPN	SCAMP3
**11**	CTSZ	HERC4	FGL1	CPSF4	**511**	SORD	ZFC3H1	SAA4	ITPA
**12**	PLA2G4C	ANKRD42	CES4A	SNF8	**512**	SLC6A8	RNF168	GSTK1	PALMD
**13**	KRT18	NA	PDK2	CCDC86	**513**	TNIP2	SCML1	HNRNPR	C7orf26
**14**	IFI35	ARMC9	CYP2C9	COPS6	**514**	FCN2	LRRC8D	ZNF462	RPL23
**15**	PIGS	DNAJB4	SERPINA4	UBE2M	**515**	HSPB1	CAAP1	DCP2	ADM
**16**	WEE2	ANKRD6	GSTA1	PHB	**516**	VASN	PBRM1	KNG1	PYGB
**17**	HM13	SATB1	UGT2B10	GNB2	**517**	CDKN1C	JMJD1C	PCP4L1	GPR89A
**18**	IGSF21	ANKRD26	TM6SF2	DHX37	**518**	TOMM34	ETAA1	VWA1	VPS25
**19**	CYP3A7	FAM200B	SEC14L2	SLC6A8	**519**	UBE2F	LEPR	BDH2	STARD3
**20**	CFB	GAS2	CRP	ABCF2	**520**	CRACDL	ANAPC1	MDFIC	YKT6
**21**	A4GALT	KCNJ10	ABHD15	ANGPT2	**521**	STX5	WIPI2	YRDC	NDUFS6
**22**	PLAAT4	USP33	HRG	RALA	**522**	TFEB	ECPAS	PCCB	RPLP2
**23**	POLD3	VPS26A	FLT1	PTCD1	**523**	KLC3	ZFP90	VMP1	PITRM1
**24**	AIFM2	RND3	AMACR	COPZ1	**524**	PRAME	SLC1A3	BRAF	FAM98C
**25**	PHC2	ZMYM6	C1QTNF3-AMACR	RUVBL1	**525**	KLLN	CARD8	KCNJ2	DTD1
**26**	C2	USP53	PCK2	BUD31	**526**	TAP2	RBM41	HSPA14	NR2C2AP
**27**	TMEM88	ZNF721	ELFN1	PKIB	**527**	TEKT5	ATP10D	CSNK1A1	DTNBP1
**28**	SECTM1	MERTK	LOX	POLD2	**528**	C1QA	LGALS4	PRDX6	POLR2L
**29**	TMIGD1	ERCC3	MED30	PPIA	**529**	RTN1	RABGAP1	SLC1A1	SLC10A3
**30**	ABCF1	WDR11	C16orf87	EIF4H	**530**	KCNF1	CREBRF	PDK1	HAS3
**31**	LTBR	RNF180	ARMCX3	PGAM1	**531**	ADSS1	TAS2R5	ETNK2	SMS
**32**	CDNF	HECTD2	PPL	PRPF19	**532**	RBCK1	ZNF776	HACE1	FBP1
**33**	PRSS21	ODF2L	S1PR5	CDK4	**533**	NLRP14	GANC	ATP6V1G1	C12orf43
**34**	PNPLA1	ZDBF2	SLC20A2	DTYMK	**534**	MTHFD1L	RCHY1	NCAM1	CHCHD1
**35**	TNFRSF1A	C10orf88	SERPIND1	DDX54	**535**	ABCF2	ZNF316	FBXL7	NXT1
**36**	VWCE	LCA5	IFITM2	NME1-NME2	**536**	DDX23	FGF2	MLYCD	GSTM3
**37**	CYTH2	RUFY2	CES1	LAGE3	**537**	NOX1	TMEM241	RPL34	REEP4
**38**	THOC6	OSBPL9	OTUD1	SEC61G	**538**	ZNF98	ZBED1	HNRNPK	UTP20
**39**	SERPING1	OSER1	CYP8B1	TXNL4A	**539**	UBE2M	GABPA	LBP	SLC2A9
**40**	THNSL1	PDE8A	UROC1	SARNP	**540**	LILRA2	ZNF214	FLOT1	TIMM9
**41**	SCUBE1	BCL10	PXDN	TPI1	**541**	CLDN4	HNRNPA2B1	CYP4F2	NT5C
**42**	PLTP	WEE1	SLC26A1	MYL6B	**542**	CFAP69	CTTNBP2NL	PCDHGA3	CDT1
**43**	CD99	CACUL1	TF	CIAPIN1	**543**	GAL3ST4	PIFO	PCDHA3	RRP36
**44**	CFL1	PPM1K	CHSY3	NME2	**544**	FKBP1A	ZNF621	COL27A1	VBP1
**45**	CCN1	ZNF25	PDE11A	MRPL11	**545**	TGIF1	FAR1	VTN	DSC2
**46**	RHOG	MTAP	SMNDC1	RPL22	**546**	CRYAB	RBM7	RORC	STC2
**47**	RNLS	PPM1D	HEY1	ATP1B3	**547**	TXNL4B	RSPH1	SNX9	NUDCD3
**48**	PLVAP	CHAC1	TERF2IP	EGLN3	**548**	MIIP	ZNF260	ATP6V1C1	ADCK2
**49**	CNKSR3	TIAL1	BCL2L11	KDM1A	**549**	FAM174C	PCDHB4	MCAM	SPATS2
**50**	DDX19B	EFCAB7	GGCX	SNRPD2	**550**	SBK2	EMC9	GPCPD1	RPS11
**51**	VPS4A	NBPF3	ATP6V0E2	SNRPD1	**551**	TP53INP2	ZBTB34	TTBK1	RPA3
**52**	HHIP	HDAC9	HNRNPH2	PTGFRN	**552**	CD151	MBD5	MRPL17	VAPA
**53**	CIB2	PIK3CA	TSKU	MAPKAPK5	**553**	PLEKHO2	TMPRSS12	IFITM3	MYBL2
**54**	CCL26	IGFBP4	KLKB1	ALG1	**554**	ADPRS	SF3A3	HACL1	KRT10
**55**	DMGDH	ABRAXAS2	SERPINA5	TIMM22	**555**	PCP4	NAALAD2	MAOB	POLR2J
**56**	ANGPTL3	AGL	SLC6A12	EIF1	**556**	RASGRP4	MAPK8	HECTD1	HNRNPR
**57**	MMP2	PTK6	ICOSLG	YWHAG	**557**	HMGA1	HBP1	ZNF217	UBE3C
**58**	CHMP7	FMR1	FNDC3B	ESYT2	**558**	INAFM2	ZNF549	BCO2	TTC27
**59**	MTARC2	TCTN2	GPD1	RPL27	**559**	RNASET2	RBM17	PRPF18	SSU72
**60**	STK19	IGFLR1	BHMT	POP7	**560**	EPGN	KLF3	SEC11A	RPSA
**61**	RAB20	PTAR1	XDH	MTCH1	**561**	INAFM1	L3MBTL3	PRMT6	RAB5B
**62**	TEAD4	ZBTB5	IVNS1ABP	KNG1	**562**	PCBP1	KLHDC7B	HMGN5	MIIP
**63**	GNG4	PARG	SGPP1	PPP2R1A	**563**	MAP2K2	KLRF1	CHAC2	RNF216
**64**	TRIP6	KCNN4	GCK	MTFP1	**564**	ZNF296	RPAP2	NUP50	QPCTL
**65**	RASD1	SLC38A2	TBC1D15	NLE1	**565**	RETREG3	TTC14	CDC14B	TMED1
**66**	ACTG1	ZNF613	ACE2	PARK7	**566**	TOE1	ABI1	FOXF1	CTDNEP1
**67**	LRRN4	THSD7A	EFNB2	ATIC	**567**	GIPC2	UBQLN1	AZI2	HPX
**68**	RELB	RAB26	DIO1	MLST8	**568**	CPA1	STX16-NPEPL1	BTF3L4	UBE2A
**69**	AFM	PACRGL	FER	HK2	**569**	IFITM1	LEMD3	NDRG2	CDC123
**70**	GPR143	UNC93A	SAP30	ATP6V0C	**570**	TAPBP	N4BP2L1	TOX2	ZBTB8OS
**71**	ST13	ZFAND4	KLC4	PLIN3	**571**	SMARCAL1	IFIT1	CLPTM1	RNF11
**72**	LRRC8D	NUP54	TGFBR3	AATF	**572**	PPT2	NUFIP2	ARL4C	NR1H2
**73**	TEKT3	EXOC1	NUDCD1	CUEDC2	**573**	MARS1	LSM11	FTCD	TRNAU1AP
**74**	CDK2AP2	BICC1	SMC4	SLC4A2	**574**	CELF4	STX12	CLNS1A	ATP6V1F
**75**	CDC20B	AK3	IMPA2	NSUN4	**575**	KMO	ZNF248	HNRNPU	NCL
**76**	SLC39A7	BMPR1A	DNAJC25	PDAP1	**576**	SKIDA1	SCAF11	ABHD14A-ACY1	PATL1
**77**	RPEL1	WASHC2A	NPM1	DNAJC7	**577**	TMEM121B	PGBD1	OGDHL	TSPO
**78**	FAM167B	PTPDC1	KLHDC7A	CCZ1	**578**	GGH	FBXL13	TSN	PWP1
**79**	HSPA5	UHRF2	DAO	PFKFB4	**579**	CYBC1	RFXAP	WNT11	BZW2
**80**	HEPACAM	IRAG2	RABGGTB	SNX17	**580**	LRPPRC	ZNF154	DNAJC9	RPL23A
**81**	CLDN9	PRPF4B	FAM89A	RHOC	**581**	ALDH9A1	EDIL3	BTBD11	MED24
**82**	ALB	WAPL	GCOM1	MRPL37	**582**	IER5L	ZDHHC12	PHF8	RPF1
**83**	CTSE	BHLHB9	FABP3	MBOAT7	**583**	ATF3	BST1	NR1I2	CENPM
**84**	BAX	MINDY3	HIC1	FAM50A	**584**	SLC35A2	DCAF12	WHAMM	SMOX
**85**	C5AR1	TCTN1	CYP4F11	SNX8	**585**	TRAPPC1	KBTBD2	PGM1	ATP6V1E1
**86**	IL32	MPHOSPH6	EGLN3	UTP18	**586**	RNPS1	TYRO3	NUP107	SMARCE1
**87**	USP51	SKIL	FAAH2	PGK1	**587**	ATP5MGL	TFCP2	CD80	GTPBP8
**88**	PDLIM1	ZNF304	SLC17A3	LSM4	**588**	CNN2	NCK1	ALPL	TMEM14B
**89**	PAH	USP54	DEK	POLE4	**589**	IFFO2	CPED1	SOCS2	ARF5
**90**	MSH3	ZNF254	MCMBP	CTDP1	**590**	PHLDA2	FNIP1	ARMCX1	ATAD3A
**91**	FLOT1	RASGRP3	CRAT	MRPS12	**591**	S100A11	ACVR2A	TIMM17B	RRP8
**92**	GSTA2	MARCHF5	UGT2B4	EFTUD2	**592**	MAD1L1	LRRC1	PCDHA7	CENPN
**93**	FGFBP1	ZZZ3	ACOX2	BRI3	**593**	HLA-B	SECISBP2L	PUM2	BAK1
**94**	GPR89B	TDRD6	KCNE4	TMEM141	**594**	UGT2B7	CENPC	CLDN5	CRTAP
**95**	ACTB	NDUFAF7	TCP11L1	DCTN2	**595**	IER3	MINDY2	SLC15A1	TRAIP
**96**	GPC4	SLF2	DPP4	KTI12	**596**	FAM110A	PLD1	RPSA	MPG
**97**	ALDH3A2	CD200	THRSP	PGLS	**597**	GABRB2	ZNF711	LRIG3	MAP4
**98**	WAS	CCP110	CES2	MARCKSL1	**598**	IL17D	LIMD2	ALDOB	STAM
**99**	IVL	IKZF5	DCTN6	MAPK3	**599**	ABCB1	RCL1	RPIA	HNRNPM
**100**	IFITM3	RASGEF1B	BLMH	DCTPP1	**600**	XPO7	SCHIP1	KIAA0586	WAC
**101**	PDIA3	LRRN2	SELENBP1	TMEM109	**601**	TMEM106A	LOC344065	NAP1L5	MLX

Table S1; Screening gene list by ICGC datasets.

Rank	Kideny Normal	Kidney Tumor	Liver Normal	Liver Tumor	Rank	Kidney Normal	Kidney Tumor	Liver Normal	Liver Tumor
**102**	ITGB2	PDCD4	NRBF2	RSL1D1	**602**	TMEM51	ZNF441	DDX24	MRPL42
**103**	COL19A1	PAWR	MOGAT2	RPS8	**603**	NA	SLC7A6OS	CLRN3	CDK2AP1
**104**	UBALD2	CDC14A	NRP1	BLMH	**604**	DNTTIP1	MAK	HNRNPA1	GRN
**105**	DCTN2	KIFBP	SULT1A2	AGFG1	**605**	ITPRIP	CCSER2	RNF13	TMEM234
**106**	GCH1	ZNF217	HNF4A	ATP6AP1	**606**	CAPG	ZNF528	ACBD4	GNS
**107**	CLCF1	GPRASP2	HADH	TBC1D7	**607**	BHMT	CEP290	XPNPEP3	C20orf27
**108**	RNF212B	RASA2	AQP7	COASY	**608**	ACTN1	DYNC1LI2	PYROXD1	LIMS1
**109**	CTSV	CDK13	LDHA	UQCRH	**609**	COL23A1	IL1RAP	NHLRC1	SNN
**110**	ACTR1A	ARHGAP12	HPRT1	PFKM	**610**	GLYAT	CDC37L1	RPS16	OAZ1
**111**	CCDC137	ZRANB2	FAM186B	KHDRBS1	**611**	DPEP3	ZNF330	PAG1	PPM1G
**112**	FADS3	DNAI4	USP2	NUDT1	**612**	GRIPAP1	MYSM1	UGP2	DVL2
**113**	ACOT6	TBC1D12	HNRNPF	UBE2S	**613**	MACROH2A1	MYLIP	TMCC3	DCBLD2
**114**	TRIM10	SMNDC1	CCT8	GTF3C6	**614**	BEX1	THNSL1	ARPC5L	ACACA
**115**	SPI1	IFNAR2	GLIS3	EEF1E1	**615**	ACOX2	NHLRC2	ACY1	ZNF540
**116**	ZYX	INHA	F10	AP4M1	**616**	CPLX1	ITGB1	TAF12	RAB41
**117**	MAP1LC3A	ARID4B	ALDH5A1	NDUFB2	**617**	CYB5A	KCTD10	UBE2D1	XPNPEP1
**118**	SH2B2	TNKS2	PAFAH2	UBE2Z	**618**	LILRA1	HGH1	RSL24D1	RPP30
**119**	ADAMTSL2	TBC1D9	AFM	PPP2R3A	**619**	C17orf64	TDRD7	LCORL	IFT57
**120**	NUAK2	PPP3CB	TMPRSS6	MARCKS	**620**	CCDC96	PCGF5	FKBP1B	SERPINB6
**121**	VSIG10L	ZNF506	FLOT2	RAMP3	**621**	TREM2	BIRC2	WWTR1	SEH1L
**122**	ARF6	HDX	UBA2	MKRN1	**622**	RHOB	POLR3A	HELB	PRMT5
**123**	PDXP	SRSF11	GTF2E2	PPP1CA	**623**	SERPINE2	EMC10	IPMK	F9
**124**	EPHA2	RPGRIP1L	MTUS1	PACSIN2	**624**	GDF5	TRUB1	CD93	SMPD4
**125**	CLDN6	TRAF3IP1	AGBL4	RANBP1	**625**	TSPAN1	SOCS6	PHGDH	EIF4EBP1
**126**	ELF3	RANBP9	MED28	DNAJB6	**626**	SRPRA	PLEKHH2	STX6	SERPINA6
**127**	SPAG4	ZFP3	AZIN1	RPF2	**627**	ULBP2	RAP2C	SARDH	POMGNT1
**128**	PILRA	MORC3	STC2	RPL11	**628**	MYC	ZNF749	SERPINE2	MRPS30
**129**	CDC25B	TUT4	SLC6A1	SNRPB	**629**	TMEM268	GOLT1A	ALDH1A3	RPL27A
**130**	SMCO4	RAB28	RFX3	MRPS7	**630**	CHRD	ZBED5	SEMA4B	ALDOB
**131**	KDELR1	MAP4K3	OSBPL1A	UBE2I	**631**	ASB10	GORAB	ALKBH8	RUFY1
**132**	IGFLR1	PKN2	UGT2B7	GNA12	**632**	OPA1	PDHX	POLA1	SNRPC
**133**	LPCAT1	ETFBKMT	PHYH	GTPBP4	**633**	YTHDF1	SLC24A1	IVD	CISD3
**134**	SCN1B	KLHL24	SH2D1B	TAF6	**634**	RIDA	CEP170	RPS23	SMPD2
**135**	PCDH15	SH3GLB1	METTL7A	CDK5	**635**	CD81	C18orf54	FBXO2	DDOST
**136**	GGT5	SPATA4	IDH1	C3	**636**	EPM2A	ME1	TSPO	EXOC3
**137**	ABHD11	RGPD6	MMACHC	VAT1	**637**	LYPLA2	TLE5	HTRA1	TK1
**138**	PDE10A	LCOR	CPN1	NOMO1	**638**	DDO	ZNF283	SCAMP1	UBXN6
**139**	PSD2	MICU3	IL12RB2	SLC38A3	**639**	LRRC37A3	CPNE8	XPOT	EMD
**140**	TTLL13	PDCD1	FGB	GLS2	**640**	PNCK	CROT	ACTR3C	PDRG1
**141**	CDC42EP1	ZNF518A	AQP11	CIZ1	**641**	UBA7	C8orf48	TLK2	SYF2
**142**	TUBA1C	RAB11FIP2	SEC61G	CLIP2	**642**	LTF	YPEL2	MECOM	SENP3-EIF4A1
**143**	HPCA	CDK6	SOCS5	C1orf174	**643**	PRSS36	NPAT	PPP1CB	XRCC6
**144**	SNAPC2	PTPN12	IDH3A	AGTRAP	**644**	SYTL3	ZNF135	CYP4F12	ADSL
**145**	SLC2A8	SGMS1	PYGL	SDF4	**645**	SIGLEC1	BLZF1	GPR153	STX5
**146**	GYPA	RAB40C	HNRNPA3	PRR13	**646**	VCP	RNF4	CCT6A	SCAF1
**147**	STRIT1	ZMYM4	KLRC3	AFM	**647**	SLC12A6	CMTR2	DNAJC21	GOLM1
**148**	RUNDC3B	IFRD1	SLC22A1	PKN1	**648**	YBX3	MYBL1	ADH4	GGCT
**149**	SIAH2	TBC1D15	EIF4E	TRIM28	**649**	WDR38	FSHR	MTHFD2L	CD47
**150**	PLEKHA8	C1GALT1C1L	TPMT	PARD3	**650**	CCDC102A	TTC26	SLC19A3	PAFAH1B3
**151**	A1CF	SENP6	PALMD	SRSF9	**651**	PDIA6	RGL1	ADAMTS9	WDR72
**152**	FAM214B	RWDD3	CLYBL	LDHA	**652**	LILRA6	SMC5	CBS	AKIP1
**153**	IQCD	LAYN	C9	ALDOA	**653**	SPINK2	ACAP2	RLIM	ST6GAL1
**154**	ADH6	MARCHF8	RABAC1	MANBAL	**654**	RBM42	ZNF257	JTB	ANAPC11
**155**	ALDH3A1	NT5C1B	GGH	AIMP2	**655**	APOBEC3A	DIXDC1	NCKAP1	CAT
**156**	DOHH	LDB1	FNDC5	UBXN1	**656**	PSPH	POU6F2	NCALD	ORC6
**157**	SPON2	WDSUB1	RAB15	BTF3L4	**657**	NCL	SP100	DPYS	B4GALT3
**158**	P2RY6	TIMM23B-AGAP6	DEGS1	PPP1CC	**658**	EFEMP2	ACSM6	HOMER3	IGF2BP3
**159**	MAN1B1	PPP3CA	IL13RA1	CHCHD6	**659**	C7	LEKR1	IGFBP3	PTMA
**160**	ENPP3	RAP1A	PGF	MZT2A	**660**	MFSD10	PPWD1	PTTG1	PIGS
**161**	CALR	MFSD14A	KANK4	NUP93	**661**	SLIT3	CRBN	ARRDC4	AP2B1
**162**	GTF2F1	MED17	SERPINA10	POLD1	**662**	ZNF428	KIAA1586	GADD45B	RPL36A
**163**	EEF1A2	ANKRD13C	SRSF3	LSM12	**663**	SMOX	RABGAP1L	GIPC2	LLPH
**164**	IQGAP2	MIER1	ALDH1B1	CALU	**664**	DRC7	AK9	RYR3	CD63
**165**	HPD	WASHC2C	TIFA	AARSD1	**665**	CST3	FZD6	OSTF1	RBFA
**166**	CCN2	FBXW2	CLU	EIF3F	**666**	ATP6V1G2	BRCC3	PHACTR4	RPA1
**167**	REC8	CCDC90B	TCEAL7	ACLY	**667**	P3H3	ZNF684	NHSL1	KPNA6
**168**	ASPHD1	ZNF644	TCEA3	RPL35A	**668**	ADAMTS16	NEK11	NGRN	TTC39A
**169**	GPR89A	SLC18B1	PGRMC2	GNB1	**669**	SLC3A1	ZNF529	CADM1	FAM120C
**170**	GPX2	ZDHHC21	LHPP	PLOD1	**670**	PDIA4	STX17	SLC13A5	PRPF31
**171**	GSTP1	WFDC2	DHRS13	DCTN1	**671**	GPKOW	KMT5B	ARMC6	TMEM98
**172**	BIK	LIG4	ANKS4B	ATP9B	**672**	EGLN3	PTPN20	DNAJC28	HNRNPUL2
**173**	HSPB8	PPM1N	FAM13A	RBM42	**673**	FBXO7	PLEKHA3	AMOT	LEAP2
**174**	FLOT2	CCDC50	SLC2A3	RPS15A	**674**	FXN	ZNF566	SYN1	MCF2L
**175**	TTPA	SRSF10	BDH1	RNH1	**675**	MANF	CGAS	SOX7	TNIP2
**176**	CEBPD	MAGEE2	JAKMIP2	POLR3K	**676**	RRAS	WDR37	GMPS	TSN
**177**	KRTCAP2	UBXN2A	PLEKHA3	BCL2L12	**677**	GADD45B	YY2	AVL9	RNF187
**178**	CTSD	BEX4	CDK17	LRRC59	**678**	HILPDA	MIS18BP1	SRPK2	VPS4A
**179**	C1QB	TMEM30A	AHSG	CSNK1D	**679**	APOH	PCDHB12	MXRA5	LIMK1
**180**	RNASE1	MARCHF7	BAALC	RABAC1	**680**	SLC10A2	RXFP2	PUS10	PRDX5
**181**	PEDS1	ZNF44	ADM	HDAC1	**681**	CRELD2	STRIP1	HMGB2	S100A9
**182**	STBD1	TANC1	MIA3	HDDC2	**682**	LAMC3	SYTL1	PDZK1	RNF220
**183**	HPGD	LURAP1	RASIP1	NOSIP	**683**	RCOR2	ZBTB21	ZNF460	SLC39A4
**184**	SLC44A4	STX2	ANGPTL2	PPP1R8	**684**	CYP4F3	ATAD1	MRPL3	MIB2
**185**	C1QC	FAXDC2	SASH1	PRMT1	**685**	CHMP4B	NAB1	NECAP1	KPNA4
**186**	RTTN	KDM4C	PGM2L1	ZNF775	**686**	SVEP1	DGCR6L	EEF1B2	CCL14
**187**	SPRYD3	MORC4	ANLN	TTC26	**687**	MPV17L2	REEP3	RRP8	MCRS1
**188**	VPS33A	ZNF451	EFNA1	TIMM17B	**688**	RAE1	CYTL1	RPS25	CLDN11
**189**	PIGR	IFIT5	NT5DC1	MRPL10	**689**	ZFP36	MASTL	LRRC3	SLC7A2
**190**	KATNAL2	ZNF17	PGLYRP2	ZCCHC17	**690**	R3HDM4	RIC8B	LSG1	P2RX5-TAX1BP3
**191**	ARL8A	OBI1	CAPN5	TMEM101	**691**	TWF2	SNRPN	RBBP9	GALNS
**192**	PPFIA3	EVI5	KCNE3	FAF1	**692**	BBS12	CAB39L	SPRED1	GLYATL1
**193**	S100A1	CAPS2	ZNF22	SLC9A3R1	**693**	PISD	SIKE1	NME2	ZNF593
**194**	RAB4B	CCL25	SLC17A1	AAMP	**694**	B3GALT6	JRKL	SLC39A11	DNASE1L3
**195**	STX4	AMMECR1L	SLC1A4	RAB1B	**695**	GLUD1	ACSS3	HNRNPL	UROD
**196**	BHMT2	RYK	MCCC2	HSDL1	**696**	GPR162	SLC25A24	LCAT	COPS8
**197**	EIF4A3	CEP85L	LRIG1	UXT	**697**	CAPN3	SAMD9	RPS18	RAB13
**198**	ENG	PSIP1	RDH5	EIF2B5	**698**	RGS9	TEKT2	NSRP1	EIF4A1
**199**	VAT1	RGPD5	KBTBD2	TBRG4	**699**	S100A2	PPP6R3	HAO2	TBL1XR1
**200**	TAX1BP3	HACE1	ACACB	PSRC1	**700**	SLC9A2	RAB25	RBM15	PUSL1
**201**	GATA6	CRIM1	FAM102A	SND1	**701**	WDR72	CAND1	RPS6	FUS
**202**	LRRC61	ZBTB1	ADH1A	RAC1	**702**	A2M	ZNF665	TPCN2	IGFBP6

Table S1; Screening gene list by ICGC datasets.

Rank	Kideny Normal	Kidney Tumor	Liver Normal	Liver Tumor	Rank	Kidney Normal	Kidney Tumor	Liver Normal	Liver Tumor
**203**	FAM50A	EPB41L2	RDH14	MZT2B	**703**	ANKRD46	KCNJ16	KCNT2	RPS7
**204**	NECTIN2	TENT5A	FAM3C	SCRN1	**704**	CORO7-PAM16	SWT1	TOP2A	ZC3HC1
**205**	PUF60	MROH6	SLC38A3	PFDN5	**705**	NRSN2	BNIP2	MGAT4A	CANT1
**206**	CBX4	PIH1D2	MAPK14	DBNL	**706**	RET	RNF133	TFDP1	STARD7
**207**	SAR1B	ZNF326	SOAT1	CDK14	**707**	PXDC1	POLK	SHROOM4	PPP1R7
**208**	SLC52A2	ZNF639	WNT5A	ARFIP2	**708**	OSCAR	ASCC1	FAM83G	PSMD13
**209**	C5orf47	FAM76B	BRMS1L	CLPP	**709**	ZNF556	GCC2	PHLPP1	GIT1
**210**	TGFB1	ZNF461	VEZT	RPS9	**710**	ANKRD53	CELA2B	FGFR1OP2	RPL10
**211**	LKAAEAR1	FCHO2	TMEM82	RPL17	**711**	NANS	ZNF568	TMEM14C	RNF10
**212**	JUN	ZNF131	SNAPC1	BRD9	**712**	SMPDL3A	ERMAP	GNL3	GLG1
**213**	EDEM2	FSCN3	RAB26	COMMD9	**713**	FAM83D	CREB1	ZMYND12	CNTD1
**214**	APOD	FBXO11	CA14	PSMD3	**714**	ICAM1	RBMS2	PTPN12	ANP32B
**215**	STAU1	PTPN7	C1orf112	ZNHIT3	**715**	PTOV1	MCMBP	PKD1L3	DFFA
**216**	SHISA5	PRKD3	ECHS1	AMD1	**716**	RRBP1	ZNF33A	LSS	BAX
**217**	IYD	EXTL2	MAMDC4	LOX	**717**	FIBP	LRRC40	LEPROTL1	NECAP2
**218**	RORC	XPA	NUDT6	AP1S1	**718**	TFE3	NUP153	NCAPG2	MMGT1
**219**	LPCAT3	CNEP1R1	AGTR1	KDELR1	**719**	ARHGEF19	IPP	SDHA	RPUSD1
**220**	NFKBIE	TRMT13	ITGB1	MLLT3	**720**	EIF4ENIF1	STK38L	SRPRB	PPP5C
**221**	GARS1	PTPRD	PLSCR1	MIF	**721**	FBXO9	SPTY2D1	BCAM	SLC25A19
**222**	PDGFB	ZYG11B	BTD	SF3A1	**722**	UBE2L6	LUC7L2	MAD2L1	CCDC59
**223**	ODF2	RSRP1	SLC25A42	BNIP3L	**723**	TBL2	POLD3	CEP170	S100A10
**224**	CNDP1	ZMYM1	OTC	RPL24	**724**	FAM149A	GGTA1	TRIM23	ABCA9
**225**	RPL28	ZBED2	PNPLA4	ALKBH4	**725**	RGS14	RSF1	C2orf49	ATP6V0B
**226**	FBLN5	IFT88	SLC10A1	GLTP	**726**	MAP3K6	PLK2	TMEM87A	MED27
**227**	KPNA2	ZNF431	RAPH1	HMGA1	**727**	HLA-DRB1	DR1	PSPC1	MIF4GD
**228**	HACL1	STAM	CCNH	PSMD9	**728**	CDH5	VMP1	SAA2	TRDMT1
**229**	AOC3	ADAMTS3	CYP2A6	GPD2	**729**	PLOD3	GOLGA1	SLC2A1	POMZP3
**230**	MAP7	CCDC82	NIT1	CHCHD2	**730**	EGR1	SPRING1	OSGIN1	C18orf54
**231**	DNASE2	MSL3	AFMID	C4orf3	**731**	SLC35C2	TSPYL5	FNTA	AGPAT1
**232**	MPC1L	ST7L	RPL31	SSBP1	**732**	C11orf54	CBFB	SLC30A5	DDIT3
**233**	EDN2	AGTPBP1	SAA1	LYRM4	**733**	STON2	PUM2	PCDHA4	CNOT1
**234**	MAPK10	ABITRAM	THSD4	SNAPC1	**734**	URM1	CCNY	PCDHGB4	REXO4
**235**	SHFL	MCL1	PTPN2	SPAG4	**735**	NUGGC	TCTN3	PNPO	RPL37A
**236**	BCL2L14	GPR137C	FGA	FLYWCH2	**736**	RCN3	WAC	NDUFA4L2	METTL2A
**237**	CKAP4	NOC3L	RIOK2	TRAPPC3	**737**	SHKBP1	NRAS	LEO1	RBMX2
**238**	ROBO4	DENND4C	CBR1	THAP11	**738**	PCSK2	DNAJC11	EXOC6	TSSC4
**239**	PSME1	ZNF718	ENPP3	TBL2	**739**	TMEM214	ACE2	STAB1	ANAPC7
**240**	PRND	C1orf52	RBP4	AP2M1	**740**	NOL4	TLCD1	PRR11	ACD
**241**	ZSWIM4	PRKCI	TLE3	MAN1B1	**741**	WNT5B	MIGA1	CEBPA	ATP6V1H
**242**	KIAA0930	MTRNR2L3	F2R	ORAI1	**742**	FTSJ1	TTC17	ZNF639	CMTM3
**243**	ASRGL1	OSBPL11	ALDH1A1	RPS16	**743**	ARAF	TGFBRAP1	AGXT	MRPL28
**244**	ETV4	SANBR	FOXP1	TMEM39B	**744**	ACOT13	DHX36	KCNMB4	COL18A1
**245**	PBLD	TUBE1	LDHD	TTC4	**745**	SNX5	INPP5F	DPY19L3	CLU
**246**	HMCN2	INIP	EHHADH	IER3IP1	**746**	WNT16	GNAQ	CCDC9	SMAD2
**247**	PFN1	CDK17	BCLAF1	SLC38A4	**747**	RAB7A	CCDC65	SULF1	BLVRA
**248**	PRCC	APPBP2	FMO4	HSPBP1	**748**	DDIT4	RGPD8	DIS3	ERP29
**249**	TICAM1	TENT2	BAIAP2	FARSB	**749**	ACMSD	ZNF555	CALU	CYP4V2
**250**	PUS1	MTF2	PELI1	RBM14-RBM4	**750**	OTOGL	CLHC1	EPB41L5	TPD52L2
**251**	MX1	PLEKHF1	CDKN1B	PSMD2	**751**	CDIPT	IPO8	RIT1	ITIH4
**252**	LILRB3	CD302	MAP7D3	PPP1R14B	**752**	FARP1	PMS2	TNFRSF11B	KIF2C
**253**	MAP3K14	FASTKD1	FUT1	EME1	**753**	MAGED1	MSL2	LIMK2	RNF40
**254**	RNF167	CEP78	PEPD	CKS1B	**754**	PTPA	TCP11L2	SNRK	CYB5R1
**255**	CDK14	RFX3	GPR4	DPCD	**755**	SETD9	MPDZ	CYP2C18	TEAD2
**256**	SUMF2	MBNL2	ACSM2B	CORO1C	**756**	PTPN6	VKORC1	CAPZA2	MDH2
**257**	NEU1	DCAF16	SGTB	RNF145	**757**	ADORA1	OXR1	SLC45A3	ZDHHC18
**258**	CSF1R	CPSF6	GPD2	LSM5	**758**	CARS1	FAM76A	TMEM128	DENR
**259**	SCNM1	REV1	GPT	ETV1	**759**	SCAMP3	EFHC2	PFKFB2	GADD45GIP1
**260**	CMTM7	ZSWIM6	CX3CL1	GATC	**760**	PLD2	SRSF2	F13B	ZC3H13
**261**	CFD	PLA2G4A	ACTN1	BAMBI	**761**	ADCK1	DYNC2LI1	EOMES	CDO1
**262**	SERPINB1	RNF138	HNF1A	DNAJC8	**762**	GCLC	TAS2R4	CYP2B6	RCE1
**263**	BUD23	ZNF223	DHX15	LRRC61	**763**	CETP	CSTF2T	CDCA5	APOC2
**264**	NPIPB15	ZNF738	SLC39A1	HNRNPL	**764**	OSBPL1A	BCAS2	GCKR	PSMD8
**265**	SEPTIN4	MAP3K7	KPNA4	BRMS1	**765**	SDC3	GTF3C4	FAM3B	PPP1R2
**266**	SLC16A3	FAM214A	GCDH	CNGA1	**766**	MESP1	CD7	TMEM182	PTTG1
**267**	MTTP	CIBAR1	PKLR	CUL4B	**767**	ITGA3	VEZF1	EDEM1	ESM1
**268**	NUDT6	TAF5	SLC46A3	RBM4	**768**	KPTN	PRRG1	ZNF329	TMUB1
**269**	KLHDC8B	SNIP1	F7	ERI3	**769**	ADM	PCF11	TSG101	PYCR1
**270**	HLA-A	NFKBIB	ZNF436	TSEN34	**770**	CRYBB1	BUB3	ODC1	LSM7
**271**	SLC35C1	SOGA3	ZGPAT	RALY	**771**	VWA8	GCNT2	OSTM1	SRI
**272**	CDKN2D	TMEM170B	SLC22A7	TUBA1C	**772**	GSPT2	NAP1L5	GOLPH3	RPL7A
**273**	MVK	FRS2	TMEM30A	LAMTOR1	**773**	USP39	RANBP10	ACSL6	RPL28
**274**	NMB	GPBP1L1	ASPDH	NKAP	**774**	SLC15A3	EIF3A	SLC39A14	FIS1
**275**	PVR	ZNF597	TANC1	TOMM7	**775**	XYLB	THOC5	TNFAIP8L1	HRAS
**276**	PNKD	TMEM163	MZT1	GRWD1	**776**	MTCH1	PLSCR4	CSGALNACT2	RPL39
**277**	WARS1	CNOT2	KITLG	NUTF2	**777**	APOL1	ZNF600	PRC1	MYPOP
**278**	EIF1	BTRC	NT5C2	RBM45	**778**	CEBPB	TTC23L	PRG4	LSM2
**279**	GAS2	MTFP1	TNC	SMARCD1	**779**	TP53I3	MYNN	CREM	SLC16A3
**280**	TMEM179B	USP1	PAK2	FBL	**780**	GMPPA	GTPBP2	SLC16A6	DAZAP2
**281**	WDR4	ARGLU1	RTN4	TUBA1B	**781**	TULP2	ING3	CYP4F3	CCDC51
**282**	BPHL	PLSCR1	LMNB1	UPP1	**782**	MBOAT7	PPP1R15A	SLC16A13	ENO2
**283**	HNRNPL	ABHD14B	XYLB	SAE1	**783**	TBC1D10B	STAM2	GZMH	NAT10
**284**	COPZ2	TRIM22	TMEM139	RRAGD	**784**	SEMA4D	DENND2C	GREB1	OIT3
**285**	DEPDC7	BATF	KIF2C	DNM1	**785**	MUL1	ZNF43	SPTBN2	IFI27L2
**286**	INTS15	FAM13B	PRKAR1A	CLTA	**786**	MS4A8	UACA	CNKSR2	MRPL45
**287**	MAP7D1	BMI1	SEMA4G	SPPL3	**787**	USP30	ZNF674	NMT1	TOMM22
**288**	DDX27	MITD1	C4orf19	NDRG1	**788**	ADAMTS2	ZNF607	BTG3	RPL18
**289**	APBB1IP	HECA	SLC5A6	DDX23	**789**	TPRN	RNASEL	TNS3	DNPEP
**290**	EPHA1	TTLL11	HP	GYG1	**790**	RTL8C	ZNF610	UBE2B	ZBTB17
**291**	NMRAL1	LAG3	BPHL	PARG	**791**	TMBIM6	TMPRSS11D	TWF1	VPS26A
**292**	MFSD2A	C2orf73	DHODH	ATP6V1D	**792**	NAGK	SDHAF1	PCDHGA8	SLC39A1
**293**	CD101	SLC35B3	ORM1	BCL7B	**793**	CUTA	ITPK1	UHRF2	RPL18A
**294**	PPP1R1B	ARL5B	CAT	CLIC1	**794**	PPIF	TFAM	MPV17L	TFPT
**295**	GALNT16	PCGF3	ANO5	MPP6	**795**	PQBP1	DENND5A	SMTN	MMP24
**296**	MMP14	FUBP1	DNAH6	FLOT1	**796**	TMEM102	STIL	CFB	P2RX4
**297**	DPP3	P4HA1	CMBL	INO80C	**797**	FOXD4	DST	MRPS5	S1PR1
**298**	P4HB	CUL7	DNAJB6	RPL5	**798**	TUFT1	CCDC30	CCDC102B	ASNS
**299**	DEPDC4	SIRT1	SEMA6B	SSNA1	**799**	GJD3	PYCR3	CDKN2D	COMTD1
**300**	GLYATL1	HNRNPDL	SNAPC3	PPIH	**800**	CTSF	CARF	CASKIN2	MED10
**301**	HOXA1	ZNF440	LIMS1	CKS2	**801**	AKIP1	CLK4	GTF2H1	MRPL52
**302**	PAF1	DBR1	GSTZ1	SLC25A3	**802**	SSRP1	TRIM32	OSMR	MRPS33
**303**	CITED4	MDFIC	SUOX	SRM	**803**	SLC16A10	ADNP2	MATR3	CHCHD3

Table S1; Screening gene list by ICGC datasets.

Rank	Kideny Normal	Kidney Tumor	Liver Normal	Liver Tumor	Rank	Kidney Normal	Kidney Tumor	Liver Normal	Liver Tumor
**304**	SIGLEC7	ZNF426	SEMA4C	PSMC3IP	**804**	SFN	RIN2	IFI16	NAA10
**305**	XPO6	STXBP3	EPHA1	VAMP3	**805**	HELZ2	DCUN1D1	CYP4V2	DPF2
**306**	CA2	SPIN1	RPL35A	MTPN	**806**	BYSL	TXLNG	PCYT2	PGP
**307**	OVCH2	WDR44	GNG10	MRPS34	**807**	REEP4	SLAIN2	LDLRAD3	TRIM47
**308**	RRH	TBCEL	AK4	MRPS23	**808**	CD4	CDC42SE1	ANGPTL3	DNASE2
**309**	CYP21A2	WDR19	STC1	MRPL20	**809**	CHIT1	ZNF611	EI24	VMA21
**310**	SLC48A1	PXYLP1	EXOSC7	RUVBL2	**810**	PDAP1	CEP135	TAF1D	ERGIC3
**311**	SPOCD1	BBOF1	EXT1	VTN	**811**	TRPM3	MLLT10	KDELR3	GDE1
**312**	MUC3A	ZNF761	APPL1	PSMG3	**812**	NA	WDR75	TM9SF3	E2F6
**313**	CAMK2G	MAPKAPK5	TRNT1	TRIP13	**813**	GK3P	ZFYVE9	IFNGR1	SUPT3H
**314**	YIPF3	PANK2	TRMT11	RNF7	**814**	NUDT13	DBF4	SPP2	CCDC9
**315**	JMJD6	ZNF519	ZBTB7B	TMEM147	**815**	INSYN2B	SKP2	LAMA4	TMEM69
**316**	FJX1	GNPDA2	RAB27A	SLCO1B1	**816**	DHFR	RBM43	GALM	BAD
**317**	LY6E	PLAG1	COPS3	BOD1	**817**	CSPP1	GLIS3	PLSCR4	DYNLRB1
**318**	CNPY3	HSPBP1	AKR1C2	RNF149	**818**	RASGEF1C	SAR1A	FBXO4	GTF3C5
**319**	RGPD4	ZNF615	ALAD	CCDC124	**819**	PIK3IP1	GNG12	CSTF2T	PSMD10
**320**	MTA2	SESTD1	SLCO2B1	C1RL	**820**	HSPB6	HELLS	FAM91A1	HDAC2
**321**	FEV	SMARCAL1	RAC3	CCT7	**821**	DAGLB	GABRA3	AKIRIN2	PELI3
**322**	WDR1	ZNF175	RPL22L1	PPP2R1B	**822**	ABCA12	KCTD18	BNIP3L	AMZ2
**323**	DUSP5	FMNL2	STRADB	NACA	**823**	APEX2	ZNF333	MRC1	G6PC3
**324**	DNASE1L3	SUPT7L	FAM177A1	PLK1	**824**	ENTPD6	PARD3	SLC5A9	CA11
**325**	LUZP2	CD8B	FGFR1	CTU1	**825**	RNF152	NA	TRAPPC4	TUBG1
**326**	PPP1R16B	RBBP6	GPAM	LRTOMT	**826**	VWF	CCDC171	CRYM	RAD54L
**327**	KYNU	EPS15	ENO3	DDIT4	**827**	C1QTNF2	HESX1	RAP1GAP	SEPHS1
**328**	IFITM2	SOX11	SMO	CBX3	**828**	HERC5	CEP120	NAGS	PTGES2
**329**	TMEM39B	MOSMO	ACOX1	SSTR2	**829**	CCDC85C	CCNT2	THPO	GAR1
**330**	VWA1	ZNF432	ABCG1	RPL13A	**830**	YARS1	ZNF148	LSM10	FNDC3B
**331**	PYGL	ADGRG6	TOR1A	NUP205	**831**	C6orf47	AQP6	HAMP	C12orf75
**332**	HSPD1	NT5C2	ECI2	PEF1	**832**	NAGPA	TNFSF15	NRARP	CSTF2
**333**	PLAAT2	DOCK4	RPS29	DAP	**833**	MEGF11	SMURF2	BOK	UPF3B
**334**	CAMK2A	ANKLE2	GCLC	CCT2	**834**	SLC11A1	DGKI	CHUK	CFB
**335**	SH3BP1	SPTLC1	DDX47	NAP1L4	**835**	GGT7	ZEB2	LZTFL1	NCS1
**336**	RMND1	EXOSC10	TMEM63B	RPL19	**836**	CD276	BBX	SSB	RPS19BP1
**337**	SRF	GTF2H1	MAPK8IP1	ATP6V0D1	**837**	ARL14	SLX4IP	TMEM39A	CDKN2A
**338**	GFRA1	RIC1	TM7SF2	GPATCH3	**838**	PMP22	HERC5	DUSP22	CRHBP
**339**	TRAF2	ZRANB1	PLAGL2	STARD3NL	**839**	ZNF385A	FGD6	PRR5L	TACC3
**340**	BCL3	KLF11	SLC23A3	GHDC	**840**	LRWD1	NCOA7	SKP2	GSPT1
**341**	PCYOX1	COQ7	DNAJC12	SKA1	**841**	PYCR1	ABRAXAS1	RPL26L1	SERPINB1
**342**	CD14	SGMS2	NSUN3	PES1	**842**	DTX2	SRSF1	NCOR1	RAD51
**343**	ATG13	CDC7	CD109	DDX10	**843**	OTUD5	PGM2	ZNF385B	MRPL47
**344**	CCT6B	INVS	CLEC2B	METTL5	**844**	FOSL2	PPIL6	HAO1	BAZ1B
**345**	HCK	G6PD	NNMT	CKAP4	**845**	STX19	DNAJB14	RBMS1	MFF
**346**	IL11	IQUB	ASRGL1	TUBA4A	**846**	RANGAP1	SLC38A3	CENPF	ORM1
**347**	QSOX1	BTBD8	RAB21	TNFSF14	**847**	NUDC	PRDM5	CYP2C8	RFC2
**348**	ADCK2	SLC9A6	ESAM	RAN	**848**	AFTPH	OCEL1	BNIP2	NME6
**349**	CACNA2D2	SLC30A7	GJB1	BIRC5	**849**	EML3	WDR31	JOSD2	HDGF
**350**	TMPRSS11E	SRPK2	CHN2	CENPW	**850**	PIERCE1	BTF3L4	MCOLN1	NCAPG2
**351**	C1R	ANXA1	CELSR1	PCBP2	**851**	B3GAT3	ZNF235	ACSM2A	RPS29
**352**	TRIM11	DDX58	AP4S1	PHF13	**852**	UNC5CL	FILIP1	RGS1	GPN2
**353**	METTL13	GPATCH2	HIF1A	C1orf122	**853**	VAMP5	STARD9	OVGP1	RPS10
**354**	NEURL3	ANKRD53	HINT1	GDI2	**854**	PI4K2A	AGAP4	BHMT2	MPZL1
**355**	PPP2R1A	C1orf105	SERPINA3	SPNS1	**855**	PSMB8	ZBTB6	SYPL1	TACC2
**356**	RRP12	KLHL36	NGDN	TMEM199	**856**	MYOM2	ZNF449	IL6R	RPS2
**357**	REP15	RABL2A	RAD21	XPO6	**857**	TIMM29	ANKRD36	TMEM33	NXN
**358**	ADGRG3	FBXL2	IER3IP1	LRWD1	**858**	SNF8	SPDYA	INPP4B	ELOVL1
**359**	EFNB1	ADGRA3	EPHX2	DDB1	**859**	B3GNTL1	ZNF184	CCNB2	PLXNA1
**360**	ASPA	ZNF256	ADH6	CLSTN1	**860**	CLDN14	B3GALT9	ACTR10	CUL2
**361**	MIDN	GPR75	TPST1	WRAP73	**861**	CC2D1B	NOL8	GPR39	METTL1
**362**	UNC119	TM6SF1	GOT1	ARPC1B	**862**	SOD2	STAMBPL1	CBFA2T3	FARSA
**363**	C1RL	CSGALNACT2	TMEM45A	CCT4	**863**	OSMR	ATF2	C12orf57	WRAP53
**364**	G6PC1	LPAR6	SEC14L1	SLC39A6	**864**	CUEDC2	RAB18	TRIM37	NHP2
**365**	RNF113B	ZFX	SYBU	MCM7	**865**	BABAM2	RANBP3L	COL18A1	PDCD6
**366**	UCP1	ZNF200	PAIP1	UBL4A	**866**	CD209	ZNF41	LYNX1	DIP2B
**367**	AS3MT	LOXHD1	COMMD10	RNPS1	**867**	SRM	IFT74	POLD3	C18orf21
**368**	DPM2	EOGT	LECT2	TNPO3	**868**	CLEC11A	B3GALNT2	RIN2	KIN
**369**	KDELR2	TYR	TBX15	PHC2	**869**	FANCC	ASB2	LRP12	GNPDA1
**370**	ZMYND15	ABCA5	STS	ERAL1	**870**	SHB	METTL15	PFDN4	BOLA3
**371**	DPF3	GK5	PPP1R3B	RPL6	**871**	CAV3	FAM126B	NARF	SLC3A2
**372**	BMP1	ERLIN1	FBXO5	TSC22D4	**872**	PTGFR	PLEKHA1	ATAD3C	RUNDC1
**373**	KLHL38	ZNF493	PLEKHB2	RP9	**873**	PRKD2	OSGEPL1	YIPF1	CFHR1
**374**	BASP1	DUSP9	MSRA	NOL7	**874**	SH3RF3	ZC3H12B	PGGT1B	RPLP0
**375**	CFAP276	GAN	ST3GAL4	KIAA0930	**875**	RPS6KL1	TTL	ZFP1	ANKLE2
**376**	VSTM2B	TLCD4	PFDN2	GSK3A	**876**	REG3G	TLL1	PLRG1	RACGAP1
**377**	CACFD1	IST1	NLGN4X	APLN	**877**	PLPP2	RLF	GAS2	PHLDA2
**378**	AGMAT	ADNP	ATP2B4	DKC1	**878**	ATF5	SLC6A1	SRP14	PGD
**379**	CCDC9	RPP30	PCDH17	DIABLO	**879**	PIWIL2	SULT1C4	DDX10	NDUFA10
**380**	PLAUR	CC2D2A	SLC2A4RG	NME1	**880**	SMIM12	SHPRH	PCDHB5	ADRM1
**381**	FOLR2	ERCC6	ALDH7A1	RHEB	**881**	PLAAT5	C21orf62	KDSR	WIPI2
**382**	BCL7B	YDJC	FN3K	PABPC4	**882**	HCLS1	SGTA	THNSL1	NUP37
**383**	TMEM115	RNPC3	NOP58	LONP1	**883**	PANK1	PGM3	MT1X	AFG3L2
**384**	THADA	ZBTB49	CHSY1	RBM17	**884**	HCFC1R1	RAB21	SLC25A23	RAD51B
**385**	VPS18	PLAA	SNRPE	SRRM5	**885**	CCKBR	DPH2	TRIM26	PAF1
**386**	GMDS	DHX15	SOD2	CCDC127	**886**	HRAS	SCN11A	JAK1	SF3A3
**387**	ZP2	ERCC6L2	KLHL25	NEU1	**887**	VSTM1	DEF6	TPD52L1	CFHR3
**388**	DMRTA1	EPC1	GLS2	CDC20	**888**	EEF2KMT	BBIP1	EAF1	B3GAT2
**389**	LIAS	ERVFRD-1	TM2D3	CCT5	**889**	CLDN7	RBM18	GNA13	POC1A
**390**	RNF187	RB1CC1	AMBP	TOMM40	**890**	PARP12	ZNF736	LTV1	NUP35
**391**	ASB15	FAS	COG5	RPS3	**891**	GRIK4	ELP1	VNN3	PHF14
**392**	TYMP	SPIN4	GNPDA2	SSB	**892**	CDKN1A	CCDC89	RPE	TCTN2
**393**	C1QTNF1	CWC22	FAM149A	TMEM54	**893**	BEST4	ABHD17B	BTG1	DDX56
**394**	SOX9	ZBTB11	PHLDB2	IMP4	**894**	HABP4	LZTFL1	LNP1	NGRN
**395**	RARRES2	FBH1	TUBE1	FCGBP	**895**	FMNL1	DET1	PGS1	RNF41
**396**	DOK3	GCLM	HNRNPM	VDAC2	**896**	RGCC	FSD1L	HAUS1	CNP
**397**	HTRA3	NEK7	PACRG	KCTD2	**897**	BUD13	ZNF540	CALCRL	PTPMT1
**398**	CEP135	ZNF766	TMED9	POM121C	**898**	HBD	FBXL3	MCTP1	GPLD1
**399**	NKAIN4	AMIGO2	VNN2	ACOT7	**899**	PLAT	PTPRN2	TEX10	TRNP1
**400**	TOR2A	PIK3AP1	RHOJ	NKIRAS2	**900**	KCNK10	KBTBD3	AFAP1L1	RAB6B
**401**	PPP1CA	GPR34	GOLT1B	TIMM50	**901**	PDPN	RAPGEF5	PAFAH1B3	RAB5C
**402**	ULBP1	SPOPL	ETNK1	CLEC3B	**902**	HNRNPM	BRAP	ATL3	ANKRD27
**403**	ABCC2	MTA3	SAA2-SAA4	OTUB1	**903**	ATG16L1	ADSS2	RXRA	ARF3
**404**	INMT	PPHLN1	EIF4A3	COPG2	**904**	C10orf99	MMS22L	PRKAA1	ARG1

Table S1; Screening gene list by ICGC datasets.

Rank	Kideny Normal	Kidney Tumor	Liver Normal	Liver Tumor	Rank	Kidney Normal	Kidney Tumor	Liver Normal	Liver Tumor
**405**	KCTD2	SLC22A15	DDX50	PTDSS2	**905**	PDIA5	MECOM	CYP3A43	RIT1
**406**	CHST12	ARHGAP21	ANO1	IMPDH2	**906**	ZDHHC1	ZNF281	ARSD	C1QBP
**407**	AKAP12	SMAD5	CDK5RAP2	VPS37C	**907**	ACOD1	RANBP2	IL18R1	SLC35E4
**408**	C14orf39	CABCOCO1	GLYCTK	TYRO3	**908**	KXD1	SLC23A2	IRS1	MYCT1
**409**	SLC27A3	C21orf91	CEBPD	AURKB	**909**	SART1	NPHP1	LYRM4	IL33
**410**	KIF4B	KIAA0825	ARHGAP35	IMPDH1	**910**	FAM83G	RBM24	PCDHA13	GSS
**411**	CERS1	ZNF347	SLC25A1	ALB	**911**	FBXL5	BMP2	S100A13	ANKRD40
**412**	TM7SF3	DENND1B	SGK1	MED19	**912**	TENT5C	RNFT1	ULK4	CDKN2AIPNL
**413**	KDM8	ORC4	CYP2A7	RCC1	**913**	TIMP1	PHF20	RNF2	YWHAE
**414**	ADGRG7	TAB3	IGFALS	NPM3	**914**	CFAP65	XPO1	RPS3	RPL36
**415**	FAM241B	STAG1	C9orf72	EIF2AK1	**915**	NALF2	STXBP5	CDIPT	GBA
**416**	AEN	RAD18	HES1	TMEM14C	**916**	MDFI	NODAL	UHRF1BP1	STUB1
**417**	TMEM54	DNAJC27	SS18	PDCL3	**917**	SLC25A36	PWP1	CLDND1	PSMB6
**418**	EIF3G	IFI44	TSPAN13	RAB3IL1	**918**	C11orf96	WDR26	FETUB	TTLL12
**419**	MCM5	ZC3H12C	TTR	EIF3M	**919**	PAQR4	SLC25A36	ACVR1C	TAF3
**420**	FOXQ1	GAPVD1	ENTPD8	TMEM160	**920**	SLC27A4	ZNF182	TSNAX	TRIM24
**421**	SH3BGRL3	SVOP	UNG	RPLP1	**921**	PRKRIP1	AVL9	MED10	FAM117B
**422**	CRISPLD2	ZNF354B	RPS6KA5	UNC45A	**922**	ZC3H14	KDM5B	SCN1B	RPA2
**423**	PFKP	DCLRE1B	MYNN	DCTN3	**923**	AK1	ABLIM1	ST6GALNAC6	DGUOK
**424**	TMEM192	LAIR2	PAH	NSUN5	**924**	PREB	WDR48	CUL4B	B9D2
**425**	VPS28	SLK	SLC22A9	TIPIN	**925**	SH3BP5L	BTBD3	DOK4	XDH
**426**	NPR1	TOPORS	TESK2	NFU1	**926**	LGALS9	PRKACB	KIF20A	TMEM51
**427**	DZIP1	RBMXL1	NOL8	SENP3	**927**	USP5	ZNF792	CYP3A4	FKBP14
**428**	MAN2B1	CEP126	CPE	RBM8A	**928**	TREX2	GTDC1	PSMC6	RAB3GAP1
**429**	CXCL16	HSD3B7	TP53INP2	COX8A	**929**	KCNE5	RMI1	MARCKS	COMMD6
**430**	RNF224	NDST2	SHMT1	FCN3	**930**	PGD	C9orf72	TNPO1	NDUFA4
**431**	RORB	MINPP1	ENTPD5	SLC1A5	**931**	ANP32B	ABHD14A	CYP4A22	EXOSC7
**432**	UBE2L3	ZNF91	SYNE2	MTHFD1L	**932**	ACAD11	CHMP6	ESD	WASL
**433**	GGACT	ORC2	RPS21	SRC	**933**	IDNK	GPA33	TMEM50A	ZNF441
**434**	PRAM1	ZNF136	PEX19	KCNJ15	**934**	GALNT14	MCM9	MED20	VPS35
**435**	C3orf49	EIF2AK2	GRHPR	POLR2G	**935**	PTGS2	SGK1	FUT11	SYNGR2
**436**	EFTUD2	MOV10L1	ARL4D	PIN1	**936**	C10orf67	MAS1L	DYNLT3	RAB35
**437**	DDA1	ASXL1	TEAD2	SSRP1	**937**	ARPC1B	WNT7B	FGFRL1	ITGB5
**438**	RAB40B	PARP8	ATAD1	BMP6	**938**	PHGDH	CBWD3	SRGAP1	AMIGO2
**439**	EFNA4	TM9SF3	CDK1	B4GALT7	**939**	SLC22A4	LEPROT	NETO2	ATG3
**440**	OAT	SLC16A12	BTBD1	PROZ	**940**	NKIRAS2	CCDC68	MFSD1	CEACAM19
**441**	LMAN2	PBX1	YARS2	HAUS1	**941**	COL1A2	CELF1	REPS2	CSNK2A2
**442**	SELP	CCDC93	TCF12	FKBP9	**942**	YWHAB	EGR2	NUDT3	GNL1
**443**	ARPC4	DDX20	FGG	PARL	**943**	REN	MSH2	HNRNPA2B1	APOC4-APOC2
**444**	IL12RB2	SHLD2	NCK1	GINS3	**944**	GPR137	FPR3	DHX9	F7
**445**	CAPNS1	RAD9B	PAPPA	NPC1	**945**	ELAVL1	SUN1	PCDHGC3	ATG9A
**446**	SDF2L1	FPGT	DHTKD1	AACS	**946**	HEYL	CNOT10	BMPER	NUDC
**447**	BLMH	FAM222A	SHBG	ARL4A	**947**	OAZ3	CDKL2	RAB18	PDCD2
**448**	SMARCB1	ZBED9	BLNK	ZNF442	**948**	C4A	TRIM59	PSMD6	PFKFB3
**449**	SRRM3	ZNF778	RNF24	C1S	**949**	RPS6KA1	KRT17	PPP2R5A	SNED1
**450**	BCAP31	DPH5	PNPLA3	MRTO4	**950**	TINAGL1	ZNF530	ZNF830	ACTN4
**451**	TRABD2A	NOSTRIN	SLC25A10	NDUFB3	**951**	PRPF6	CTNNAL1	ALDH18A1	ARHGAP1
**452**	AP2S1	ZNF583	DCXR	JMJD6	**952**	PNMA5	OGFRL1	PANX2	SNRPG
**453**	NSFL1C	ZNHIT6	EIF1	MRPL17	**953**	LGR6	AFTPH	ZNF484	SMARCB1
**454**	TRH	AEBP2	APCS	C8B	**954**	G6PD	GPCPD1	CYP2J2	TLK2
**455**	CLN3	NKG7	RIF1	DOHH	**955**	MAD2L2	PDSS1	RPL5	SNRNP40
**456**	TKT	SYNGR3	MYH14	RPS27A	**956**	C4B	TPSG1	ALDH3A2	SRGAP2
**457**	DIO1	ZKSCAN7	KHDRBS1	MMD	**957**	H2AX	ABCB10	ZNF512B	MAP1LC3B
**458**	SERPINA6	ASB3	ARFGAP3	MED22	**958**	GRM8	CCDC160	SCRN2	RPS19
**459**	AIG1	OTUD1	SNRPD1	SLC6A1	**959**	MAFF	ROCK1	AQP9	NAGPA
**460**	ISM1	FAIM	NCOA1	ARMC10	**960**	POLA2	WAS	RPS12	FIP1L1
**461**	UBE2I	HSD17B12	PCBP1	CBX1	**961**	KRT80	ZNF222	ACAA1	TBC1D22A
**462**	GGT6	RAB14	PANK4	LRRC47	**962**	RFXANK	RNF2	HMGCS2	ITIH3
**463**	PYGM	ANKRD50	CRNKL1	TXNL1	**963**	TRIM39	DHX32	ANXA7	PSME3
**464**	MRO	TTC30B	RGN	ST3GAL2	**964**	SF3B2	TRA2B	AMT	MRPL48
**465**	ENPP6	NCAN	ARID4B	DYNLL1	**965**	CHRNA3	NMNAT1	NDUFAF2	NDUFA1
**466**	KLF6	MAP9	RAPGEF5	AXIN1	**966**	RTL8B	SLC5A4	CWC22	KIAA2013
**467**	TLCD3A	ZNF765	EDNRA	SPAG16	**967**	ALDOA	SEPSECS	TPT1	SLC27A2
**468**	TALDO1	CCDC186	LIME1	GAPDH	**968**	RNF175	CEP44	BACE1	PIH1D1
**469**	LRRC28	MIEN1	ARMCX6	ZNF428	**969**	ALDOB	ZNF614	RPS8	C5
**470**	PIPOX	CCDC148	MTHFD1L	PKMYT1	**970**	FAM234A	TMF1	CAST	TRADD
**471**	GPAT3	CNTLN	POLE4	MAGOH	**971**	MFGE8	CSNK1G3	CYB5D2	RNF4
**472**	SCARF2	FCRL3	PLEK2	TMEM60	**972**	KRT20	THBS1	SLC28A1	HAX1
**473**	NFAM1	UBE2D1	FCHO2	HOMER3	**973**	CDADC1	C7orf31	CP	RRP1
**474**	OLFML3	CHORDC1	MTRF1L	TFEB	**974**	IFI30	TRIO	CDH13	CREB3
**475**	PDE6B	PPTC7	UPRT	TXNDC12	**975**	ADA	BOD1L1	HMGB3	SMTN
**476**	IDH1	TSC22D2	ABCC6	EIF3D	**976**	DPAGT1	ZNF701	RPS10	OPN1SW
**477**	EIF2B1	ITSN1	RAB13	BRAT1	**977**	DDX19A	KLHL9	VPS37A	NDUFA13
**478**	FARSA	EFCAB13	CALM2	RPL36A-HNRNPH2	**978**	LSR	ARL6IP6	ACY3	EPHB2
**479**	SERPINH1	DYRK2	GUCY1A2	EXOSC10	**979**	SLC10A3	RASA1	SLC30A7	MST1
**480**	NSDHL	ATF1	NFKBIB	GALE	**980**	PRNP	NR1D2	MAML3	HNRNPUL2-BSCL2
**481**	MMP23B	RNF34	TBK1	PHF19	**981**	CCDC97	KAT6B	WARS2	RFC4
**482**	UBR4	TAF1A	AP1AR	SNRPF	**982**	APLP1	USP46	NR1I3	AP2S1
**483**	CYP4V2	MS4A7	AADAT	ISY1	**983**	PTPN18	ZNF558	HABP2	MICALL1
**484**	LCN2	ZNF345	DHCR24	C11orf49	**984**	NOPCHAP1	DCUN1D4	AS3MT	N4BP2L1
**485**	MMUT	ZNF675	UBE2S	TMEM9	**985**	NAT1	SMARCAD1	HK2	MCAT
**486**	SPINT1	APH1B	PPARA	NOB1	**986**	TGM2	TRIP12	CD28	RAB21
**487**	SMARCD2	MYO5A	TCF4	RPS5	**987**	ACD	SAMD13	CLVS1	RNASEK
**488**	NT5E	MAP3K2	PREX2	SHCBP1	**988**	CAPZB	CCDC6	SGCD	SET
**489**	JPT1	TAB2	ABCG8	NOC2L	**989**	FCGR3A	DYRK3	VEGFC	C12orf73
**490**	ACAD9	RC3H2	ADRA1B	SMARCAL1	**990**	ESYT1	MZB1	MICU1	CNPPD1
**491**	CLDN3	ZNF436	SPRY1	IGSF3	**991**	FBXO10	CNOT6	HCN3	GABARAPL2
**492**	GHR	RPGR	SEC61B	VPS41	**992**	HSD3B7	ZNF655	PTPN9	MRPL21
**493**	MT2A	BTN2A1	DHFR	SF3B2	**993**	YWHAH	N4BP2L2	CCP110	BCAP31
**494**	CHST7	TBC1D19	PRPF38B	CCT6A	**994**	SLC17A3	MLF1	MKI67	RBX1
**495**	LRRC25	SNX14	SMN1	G6PD	**995**	PPP1R14A	ZNF816	UTP6	LHFPL2
**496**	NFKB2	SWAP70	EGFLAM	C7orf50	**996**	CDH6	VGF	ATR	CSNK2B
**497**	MGAT4B	PHTF1	SMN2	EPDR1	**997**	RBM22	AP3M1	TMEM65	MRPS5
**498**	DAPK3	CDYL	GARNL3	PPHLN1	**998**	HSPB2-C11orf52	MUC1	PDE1A	CPS1
**499**	CALM1	RBM20	TSPAN9	NSF	**999**	PPIB	DIDO1	BMPR1B	SRR
**500**	PDE6H	PKP4	TBL1XR1	TRAPPC4	**1000**	NLRX1	UBE2D3	FGFR4	SPRYD3
